# Association of Renal Function and Direct-Acting Antiviral Agents for HCV: A Network Meta-Analysis

**DOI:** 10.3390/jcm7100314

**Published:** 2018-09-29

**Authors:** Chih-Chin Kao, Yu-Shiuan Lin, Heng-Cheng Chu, Te-Chao Fang, Mai-Szu Wu, Yi-No Kang

**Affiliations:** 1 Division of Nephrology, Department of Internal Medicine, Taipei Medical University Hospital, Taipei 110, Taiwan; 121008@h.tmu.edu.tw (C.-C.K.); fangtc@tmu.edu.tw (T.-C.F.); 2 Graduate Institute of Clinical Medicine, College of Medicine, Taipei Medical University, Taipei 110, Taiwan; maiszuwu@tmu.edu.tw; 3 Department of Internal Medicine, School of Medicine, College of Medicine, Taipei Medical University, Taipei 110, Taiwan; chu5583@ms55.hinet.net; 4 School of Medicine, College of Medicine, Taipei Medical University, Taipei 110, Taiwan; scottyslin@hotmail.com; 5 Center for Evidence-Based Medicine, Department of Education, Taipei Medical University Hospital, Taipei 110, Taiwan; 6 Division of Gastroenterology, Department of Internal Medicine, Taipei Medical University Hospital, Taipei 110, Taiwan; 7 Division of Nephrology, Department of Internal Medicine, Shuang Ho Hospital, Taipei 235, Taiwan

**Keywords:** direct-acting antiviral agents (DAAs), hepatitis C virus, chronic kidney disease

## Abstract

The effectiveness and safety of direct-acting antiviral agents (DAAs) in hepatitis C virus (HCV) patients with renal insufficiency remain controversial. Therefore, this network meta-analysis aims to assess effectiveness and safety of DAAs in populations with different renal function. The pooled data were obtained from Cochrane Library, EMBASE, PubMed, and Web of Science. Thirteen studies recruited 6884 patients with hepatitis C infection and reported their outcomes in relation to different levels of renal function after treatment with DAAs. The results showed no difference in the virologic responses among patients with different renal function. Regarding safety, whereas in patients without chronic kidney disease (CKD) or with early CKD DAAs were associated with a risk ratio (RR) of 0.14 (95% confidence interval (CI), 0.04 to 0.43) for renal disorder, increased risk of renal function deterioration was found in advanced-CKD patients, though this effect may be related to the natural course of advanced CKD. Similarly, patients without CKD or with early CKD showed a lower risk of anemia (RR, 0.34; 95% CI, 0.20 to 0.57) and discontinuation (RR, 0.41; 95% CI, 0.39 to 0.56) than patients with advanced CKD. The efficacy of DAAs for HCV treatment was comparable in patients with advanced CKD and in those with early CKD or without CKD. However, the safety of DAAs should be verified in future studies.

## 1. Introduction

Approximately more than 170 million patients have chronic hepatitis C virus (HCV) infection worldwide, leading to 500,000 deaths annually [1]. Chronic HCV infection can progress to liver fibrosis, liver cirrhosis, and hepatocellular carcinoma [2]. HCV patients frequently have kidney disorder, which is one of the most common extra-hepatic disfunctions associated with HCV infection, appearing in 10% to 60% of patients [3,4,5]. A Taiwan study showed that 16.5% of HCV-seropositive patients presented chronic kidney disease (CKD) as a comorbidity [6]. Moreover, HCV infection leads to increased risk of advanced CKD [7]. These patients also face a higher risk of proteinuria and glomerulonephritis such as membranoproliferative glomerulonephritis or mixed cryoglobulinemia vasculitis [8,9]. Meanwhile, in dialysis patients, the prevalence of HCV infection ranges from 2.6% to 22.9% [10], including in a US cohort showing that the prevalence of HCV-positivity in hemodialysis patients was 14.4%. This concomitant HCV infection is associated with an increased risk of all-cause and cardiovascular mortality in hemodialysis patients [11].

HCV-infected patients had a lower risk of end-stage renal disease after treatment with pegylated IFN and ribavirin [12]. A study from Taiwan demonstrated that the patients treated for HCV infection had a 84% reduced risk of end-stage renal disease, a 47% reduced risk of ischemic stroke, and a 36% reduced risk of acute coronary syndrome [13]. However, physicians are reluctant to use this regimen because of potential side effects and intolerance [14]. In addition, the response of IFN-based therapy in CKD and dialysis patients is suboptimal, with only half of the patients reaching a sustained viral response (SVR) [15]. The emergence of direct-acting antivirals (DAAs) has brought HCV treatment into a revolutionized era. The rate of SVR at post-treatment week 12 (SVR 12) with undetectable HCV RNA was over 90–95% in the normal renal function subjects, with tolerable adverse events [16,17,18]. However, the use of DAAs is limited to those patients with mild to moderate renal dysfunction. A few randomized controlled trials considered DAAs for advanced CKD patients. The phase 3 randomized trial “C-SURFER” demonstrated that CKD-stage-4–5 patients with HCV genotype 1 infection received DAAs with a 94% SVR 12 rate [19]. Kohli et al. showed the SVR 12 rates of DAAs for the treatment of HCV in CKD stages 4 or 5 was 90% to 100% [20]. However, these randomized controlled trials were conducted in highly selected patients and may not be translatable into real-world practice. A head-to-head comparison of advanced CKD and early CKD patients was not performed, either. Therefore, we aimed to systematically collect all the available clinical comparative studies for DAAs in patients with different renal conditions. We focused on the efficacy and safety of DAAs for these populations. 

## 2. Methods 

All data analyzed during this study were previously published; therefore, it this study was exempted from institutional review board approval. The study was conducted and reported according to the Preferred Reporting Items for Systematic Reviews and Meta-Analyses (PRISMA) guidelines.

### 2.1. Literature Search and Selection

We systematically identified citations from the Cochrane library, EMBASE, PubMed, and Web of Science with relevant terms of CKD, HCV, and DAAs. The primary search strategy was completed in PubMed and was then adapted to other databases. The search strategy consisted of natural language, medical subject headings, and Boolean operators without restrictions of language and time (Table S1). The final searches were completed on 23 January 2018.

Two investigators (C.C.K. and Y.S.L.) screened the potentially acceptable citations in two steps: screening of titles and abstracts, retrieving and reviewing the full-texts of potential eligible studies. The inclusion criteria of the screening were as follows: (i) comparative study, (ii) patients with HCV, (iii) usage of DAAs, and (iv) available data for different renal status. The exclusion criteria were as follows: (i) study focusing on patients after organ transplantation, (ii) patients in a specific situation (human immunodeficiency virus only or on dialysis only), and (iii) short or incomplete study information with only abstract or conference documents. Any disagreement regarding study eligibility between the two investigators was resolved through a discussion with the third investigator (Y.N.K.).

### 2.2. Quality Assessment

The two investigators individually assessed the risk of bias in the included studies by using the Methodological Index for Non-randomized Studies (MINORS) [21]. The first to the eighth items scrutinize the methodological quality of papers, and the ninth to the twelfth items are the additional criteria for comparative studies. MINORS scores are: 0 (not reported), 1 (reported but inadequate), and 2 (reported and adequate). Because the total score of MINORS is 24, in this study, the value was set to 12 (half of the total score) for poor quality and to 22 (90% of total score) for good quality. MINORS scores between 13 and 21 were judged as fair quality.

### 2.3. Data Extraction and Statistical Analysis

Data and relevant information were extracted by two investigators (Y.N.K. and Y.S.L.) independently. They identified and double-checked the information on the patients’ characteristics and outcomes; particularly, virologic responses, alanine aminotransferase (ALT), renal disorder, anemia, eruption, and discontinuation were examined. The virologic responses include rapid virologic response (RVR), SVR 12, and virologic response at the end of treatment (VRET). The results were mainly expressed as risk ratios (RR) for dichotomous data in the random-effects model. The fixed-effect model was supplemented with appendices. Peto odds ratio (Peto OR) was calculated when any zero-cell was included in meta-analysis. Effect sizes were determined with 95% confidence intervals (95% CI). Heterogeneity across the pooled studies was assessed and determined as *I*^2^, which represented the percentage of total variability across the pooled studies. Its values of 25%, 50%, and 75% were defined as low, moderate, and high heterogeneity, respectively [22]. The inconsistency between direct and indirect comparison in network meta-analysis was detected, evaluated by H-statistics, and defined at three levels, including minimal (*H* < 3), moderate (3 ≤ *H* < 6), and severe (*H*≤) inconsistency [23]. This study detected the publication bias of the meta-analysis by using Luis–Furuya–Kanamori asymmetry index (LFK) or Begg and Mazumdar rank correlation and Egger’s regression intercept. Statistical significance was set at *p* < 0.05 for all analyses. All the network meta-analyses were conducted on MetaXL, as recommended by Cochrane (http://methods.cochrane.org/cmi/network-meta-analysis-toolkit), and all the head-to-head meta-analyses were conducted on RevMan version 5.3 (RevMan 5.3, The Cochrane Collaboration, Oxford, UK).

## 3. Results

**Figure 1:**
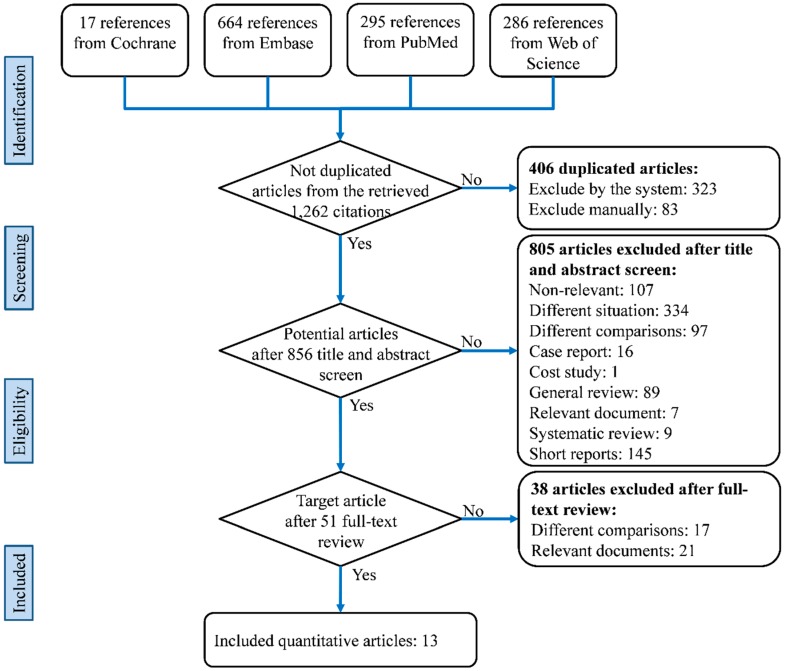
Flowchart of the systematic review and meta-analysis according to the Preferred Reporting Items for Systematic Reviews and Meta-Analyses (PRISMA) guidelines.

A total of 1262 citations was returned by all databases, of which 13 studies met the eligible criteria [19,24,25,26,27,28,29,30,31,32,33,34,35]. A flow diagram of the selection process in this study is shown in Figure 1.

### 3.1. Characteristics and Quality of the Included Studies

The identified 13 studies included 6884 patients with HCV, of which 6083 patients had no or early CKD, 611 had advanced CKD, and 190 were on dialysis. Of the 13 studies, 10 studies compared early-CKD to advanced-CKD patients [24,27,28,29,30,31,32,33,34,35], one study compared early-CKD to dialysis patients [26], and the other two studies compared advanced-CKD to dialysis patients [19,25]. The characteristics of the 13 studies are listed in Table 1. The treatments included daclatasvir (DCV) and asunaprevir (ASV), a Sofosbuvir-based (SOF) combination, and others. Seven of the included studies were from Japan [24,26,27,28,29,33,35], three from USA [30,32,34], and three from multiple regions (America, Asia, Australia, and Europe) [19,25,31]. The quality of these studies was fair to good and their MINORS score was 18.15 ± 2.94 (14 to 23) (Table S2).

### 3.2. Primary Outcomes

**Figure 2:**
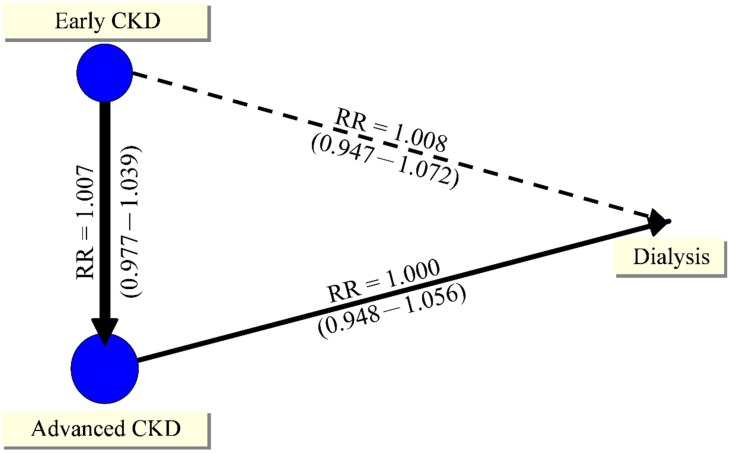
Network plot of sustained virologic response at post-treatment week 12 among patients with different renal conditions. RR, risk ratios; CKD, chronic kidney disease.

The SVR 12 data of patients with different renal function presented in the seven studies compared an early-CKD group (estimated Glomerular filtration rate, eGFR (estimated Glomerular filtration rate) ≥ 60 mL) with an advanced-CKD group (eGFR < 60 mL without dialysis), or an advanced-CKD group (eGFR < 60 mL without dialysis) with a dialysis group [19,24,27,28,31,33,35]. The network meta-analysis showed no significant difference in SVR 12 rates among patients with different renal function (Figure 2). No difference in SVR 12 was observed (RR, 1.007; 95% CI, 0.977 to 1.039) between the early-CKD group (2028/2586, 74.42%) and the advanced-CKD group (302/346, 87.28%). The pooled data of SVR 12 also showed no significant difference between the advanced-CKD group (29/29, 100%) and the dialysis group (86/87, 98.85%) (RR, 1.000; 95% CI, 0.948 to 1.056). In the adjusted indirect comparison (AIC), there was no significant difference in SVR 12 between the early-CKD group and the dialysis group (RR, 1.008; 95% CI, 0.947 to 1.072) (Figure S1). There was no sufficient evidence to support a publication bias for this result (LFK = 0.26) (Figure S2). The inconsistency test did not detect inconsistency (*H* = 1), because the network meta-analysis had no loop.

However, a further subgroup analysis of SVR 12 rates between the early-CKD group (eGFR ≥ 45 mL) and the advanced-CKD group (eGFR < 45 mL) showed that the advanced-CKD group (eGFR < 45 mL) might have a better SVR 12 than the early-CKD group (eGFR ≥ 45 mL) (RR, 0.90; 95% CI, 0.84 to 0.97). Very low heterogeneities (*I*^2^ = 0%) were observed in each subgroup, but moderate heterogeneity (*I*^2^ = 52%) was found in the total effect. This moderate heterogeneity was due to subgroup differences, because the test for subgroup differences showed high heterogeneity among the subgroups (*I*^2^ = 76%) in the random-effects model (Figure S3) and moderate to high heterogeneity among the subgroups (*I*^2^ = 68.9%) in the fixed-effect model (Figure S4).

**Figure 3:**
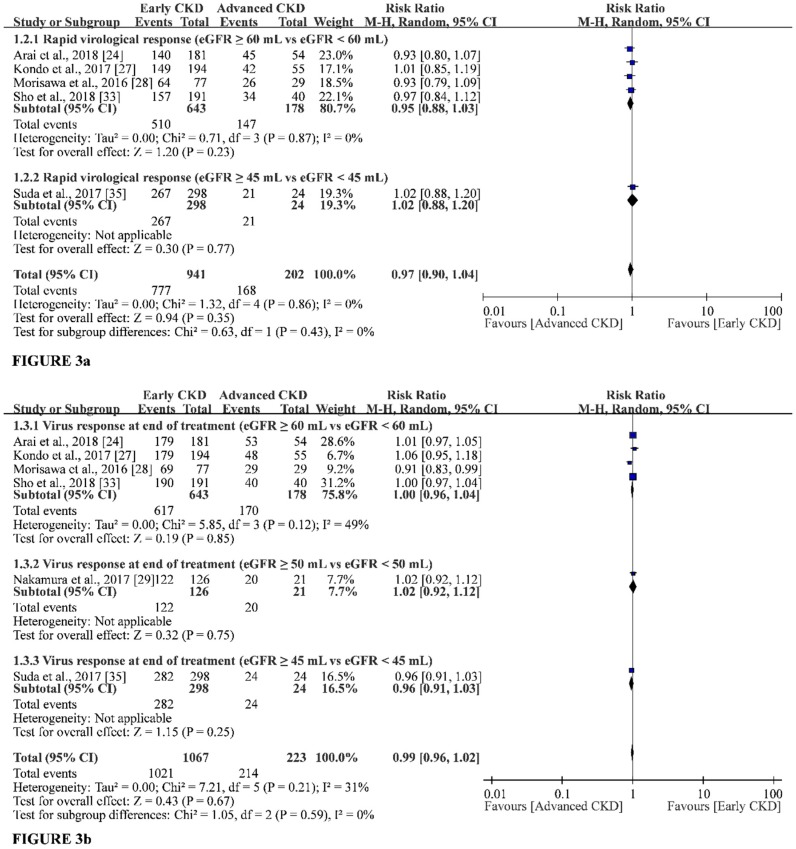
Forest plot of rapid virologic response and virologic response at the end of treatment. eGFR, estimated Glomerular filtration rate.

Five of the included studies reported RVR in the early-CKD group (eGFR ≥ 60 mL/eGFR ≥ 45 mL) and in the advanced-CKD group (eGFR < 60 mL without dialysis/eGFR < 45 mL without dialysis) [24,27,28,33,35], but no available data of RVR was found for the dialysis group. Therefore, this study could only conduct a head-to-head meta-analysis for RVR rates between the early-CKD group and the advanced-CKD group. The pooled result showed no significant difference in RVR rates between the two groups (RR, 0.97; 95% CI, 0.90 to 1.04). The heterogeneity of this result was very low (*I*^2^ = 0%, *p* = 0.86), indicating the achievement of similar results in these studies (Figure 3a). Similar outcomes could be observed in the fixed-effect model (Figure S5).

Six of the included studies reported VRET in the early-CKD group and in the advanced-CKD group [24,27,28,29,33,35]. None of the studies supported the presence of VRET in the dialysis group. Therefore, this study conducted a head-to-head meta-analysis for VRET rates between the early-CKD group and the advanced-CKD group. The results showed similar VRET rates between the two groups (RR, 0.99; 95% CI, 0.96 to 1.02), with low to moderate heterogeneity (*I*^2^ = 31%, *p* = 0.21) (Figure 3b). The fixed-effect model also showed similar outcomes (Figure S6).

### 3.3. Secondary Outcomes 

This systematic review synthesized quantitative data of safety issues presented in the selected articles, including ALT elevation, renal disorder, anemia, eruption, and overall discontinuations. Only renal disorder, anemia, eruptions, and overall discontinuations could be found in the early-CKD group and the advanced-CKD group.

Five of the included studies reported relevant information pertaining to ALT elevation [24,26,27,28,35]. The five studies compared the early-CKD group (eGFR ≥ 60 mL) to the advanced-CKD group (eGFR < 60 mL), or the advanced-CKD group (eGFR < 60 mL) to the dialysis group. The results of this network meta-analysis showed no significant differences in ALT elevation rates among patients with different renal conditions (Table 2). The pooled data showed that the early-CKD group had similar ALT elevation rates (30/750, 4.00%) as the advanced-CKD group (10/162, 6.17%) (RR, 0.730; 95% CI, 0.295 to 1.807); in addition, no significant difference was observed (RR, 1.460; 95% CI, 0.18 to 12.03) between the early-CKD group and the dialysis group. The AIC indicated that there was no significant difference in ALT elevation rates between the advanced-CKD group and the dialysis group (RR, 2.00; 95% CI, 0.298 to 13.435) (Figure S7). The heterogeneity in all the head-to-head comparisons was very low (*I*^2^ = 0%), and the inconsistency of the results was minimal (*H* = 1). 

Renal disorder events were reported in five of the included studies [24,27,31,33,35]. The results showed that the early-CKD group (24/2580, 0.93%) had a lower renal disorder rate than the advanced-CKD group (14/246, 5.69%) (RR, 0.14; 95% CI, 0.04 to 0.43). A low to moderate heterogeneity was observed in this meta-analysis (*I*^2^ = 35%, *p* = 0.20) (Table 3 and Figure S8). A similar trend was observed in the fixed-effect model with Peto OR (Figure S9).

Anemia (hemoglobin < 8–10 g/dL) was reported in five of the included studies [30,31,32,33,35]. The pooled result showed that the early-CKD group (328/5428, 6.04%) had a lower anemia rate than the advanced-CKD group (42/257, 16.34%) (RR, 0.34; 95% CI, 0.20 to 0.57). A low to moderate heterogeneity was observed (*I*^2^ = 47%, *p* = 0.11) (Table 3). This low to moderate heterogeneity was from subgroup differences, because heterogeneities in each subgroup were very low (*I*^2^ = 0%). The test for subgroup differences showed moderate to high heterogeneity among the subgroups (*I*^2^ = 69.4%, *p* = 0.04) (Figure S10). The fixed-effect model also provided similar results (Figure S11).

Relevant information regarding eruptions was reported in five of the included studies [24,27,29,30,32]. The pooled data showed there was no significant difference in the eruption rates between the early-CKD group (71/3724, 1.91%) and the advanced-CKD group (10/250, 4.00%) (RR, 0.74; 95% CI, 0.14 to 3.82). The heterogeneity of the result was high (*I*^2^ = 75%, *p* = 0.003) (Table 3 and Figure S12). Only one subgroup (eGFR ≥ 90 mL versus eGFR < 90 mL) showed a significant difference in the eruption rates between the two groups (RR, 0.18; 95% CI, 0.08 to 0.40) and was based on only one study [30]. The fixed-effect model with Peto OR also showed similar outcomes (Figure S13).

Data regarding discontinuations was reported in eight of the included studies [27,28,29,30,31,32,33,35]. The pooled data showed that the early-CKD group (439/5858, 7.49%) had a significantly lower discontinuation rate than the advanced-CKD group (48/329, 14.59%) (RR, 0.41; 95% CI, 0.30 to 0.56). The heterogeneity of the result was low (*I*^2^ = 3%) (Table 3 and Figure S14). Similar outcomes and trend could also be observed in the fixed-effect model with Peto OR (Figure S15). The small study bias was not detected in Begg and Mazumdar rank correlation (tau = 0.048; *Z* = 0.150, *p* = 0.881) and in Egger’s regression intercept (*t*-value = 0.515; *p* = 0.628) (Supplemental File 1, Figure S16).

## 4. Discussion

Our main findings indicate that DAAs for HCV infection have comparable safety and efficacy in advanced-CKD patients and in patients without or with early CKD. Advanced-CKD patients are a specific patient population, difficult to treat. Since advanced-CKD or dialysis patients are older, sicker, and with multi-comorbidities, they often have poor tolerability of IFN-based regimens. A previous meta-analysis showed DAAs-based antiviral therapies were effective and well tolerated in stage-4–5 CKD patients [36]. The aggregate study that included 11 studies reported an effective treatment with DAAs for advanced-CKD patients, with SVR 12 reaching 93%. However, this study cannot prove that DAAs have similar efficacy in patients with different renal status. Our study was performed using head-to-head comparisons between advanced- and no- or early-CKD patients, and a similar efficacy was found in these groups of patients. These results provides a strong evidence that the viral response to DAAs is not influenced by renal failure, even in dialysis patients. However, the safety analysis introduced some concern, since adverse effects and early treatment interruption seemed more common in patients with advanced CKD.

Advanced-CKD patients had a higher risk of renal function deterioration, anemia, and early discontinuation. The increased risk of renal function deterioration was not previously reported. Previous randomized trials showed that only up to 1.2% of advanced-CKD patients developed renal function deterioration after DAA treatment [19,25]. Our pooled results also showed that no-CKD and early-CKD patients (eGFR ≥ 60) had a lower risk of renal function progression than advanced-CKD patients (eGFR < 60) after DAA treatment. This result is similar to those of previous reports. An abrupt decline in renal function has been reported in advanced-CKD patients [37,38]. Our meta-analysis also demonstrated no significant difference in the risk of renal function progression between the group of patients with eGFR ≥ 45 and the group with eGFR < 45, after DAA treatment. Although these results seem contradictory, they actually suggest that CKD progression due to DAA treatment may more likely occur in the advanced-CKD population than in no-CKD and early-CKD patients. Because the population with eGFR ≥ 45 included patients with eGFR < 60, this population may also be more easily led to renal function deterioration after DAA treatment. However, whether renal function deterioration is associated with DAAs or with the baseline renal function needs to be further investigated. Anemia occurred at a frequency of 45% after DAA treatment in the advanced-CKD patients, most of which suffered from grade-2 anemia (hemoglobin 8–10 g/dL) [39]. Anemia can be controlled by the interruption of the administered drugs and the prescription of erythropoietin [39]. This result remained consistent despite removing ribavirin [31]. This was not unexpected, since anemia is frequently present in advanced-CKD. Disease itself may cause renal anemia due to insufficient production of erythropoietin [40]. Similarly, whether anemia is a result of advanced CKD or secondary to the DAA treatment needs to be clarified in further studies. 

This study showed that advanced-CKD patients were associated with higher risk of treatment discontinuation, with a discontinuation rate of 14.6%. This is similar to the results of a previous review showing a 0% to 17% discontinuation rate for DAAs [41]. Most of the DAAs’ adverse effects are non-specific, such as headache, nausea, and fatigue, ranging from 0% to 67%. The RUBY-I trial showed that patients who had CKD stage 4–5 or dialysis frequently developed a mild to moderate adverse event [39], though no patients discontinued DAAs during the study. The exact mechanisms leading to discontinuation were beyond the goals of this study. Drug discontinuation may result from combined adverse events or be related to different characteristics of the patients in the different groups. 

Few CKD patients in the real world received DAAs for HCV treatment; for example, only 6.9% of these patients received DAAs in a nearly two-year observational study conducted in the U.S. [30]. DAAs provide several benefits to CKD patients with HCV infection. One benefit is the reduction of cardiovascular morbidity and mortality, the most predominant complications in CKD patients [42]. Studies showed HCV infection was associated with increased risk of cardiovascular events. In addition, treatment of the HCV infection improved renal and cardiovascular outcomes in diabetic patients [13,43]. Other benefits of DAAs treatment for CKD patients include reducing the risk of renal function progression and liver disease progression, as well as improving patients’ well-being [44]. The evidence comes from a previous study showing that DAAs administered to patients with HCV-related glomerulonephritis achieved an 83% SVR, with subsequent improvement of serum creatinine and reductions in proteinuria [34]. 

This study has several limitations. First, it could not be designed as a randomized controlled trial, because the aim of our study was to compare DAAs among patients with different renal status. Therefore, some safety issues could not be proved. Second, our analysis consisted of several DAA regimens. Although the statistics demonstrated acceptable heterogeneity and consistency in our results, the safety issue might be influenced by the DAA regimens. Because different regimens led to DAA excretion by different organs (kidney or liver), the combined results cannot provide any recommendation for DAA regimens. Third, we could not stratify the data according to the patients’ characteristics and comorbidities, such as age, sex, HCV genotype, hypertension, or diabetes. These data points for each patient could not be obtained. In addition, it was not known whether the patients were treatment-naïve or not. Thus, we cannot provide any specific recommendation for each individual, but we can evaluate the overall outcome of DAAs. Similarly, our study could not access raw data before and after the DAA treatments, and the results should be translated to clinical practice cautiously. We anticipate a further meta-analysis using individual patients’ data on this topic in future. Fourth, only three studies provided separate data of patients that underwent dialysis. It is necessary for future studies to address this issue. Lastly, eGFR was calculated by various formulas. Reviewing these papers, six of 13 (46%) studies [19,24,27,32,33,35] determined the eGFR by the Modification of Diet in Renal Disease method, with only one of 13 (8%) [34] using the CKD epidemiology method. The other studies did not define how they calculated the eGFR. That is to say, eGFR calculations were heterogenous among the studies we examined for our meta-analysis. Consequently, our results should be interpreted carefully and be cautiously adopted in clinical practice. 

## 5. Conclusions

In summary, our study found that the efficacy of DAAs for HCV infection was comparable in advanced-CKD patients and in patients without CKD or with early CKD. However, the optimal regimen and the treatment effects on renal function progression require more investigation. The safety analysis showed increased risk of renal function deterioration and anemia events in advanced-CKD subjects, though these might be due to the natural disease progression in these patients.

## Figures and Tables

**Table:** (A)

		Inclusion	Sample Size			
Location	Region	Period	NONE TO EARLY	Advanced	Dialysis	Treatment
Arai et al.	Japan	10/2012 to	181	54	NA	Ombitasvir/Paritaprevir/
(2018) [24]		03/2017	(eGFR ≥ 60 mL)	(eGFR < 60 mL)		Ritonavir
Gane et al.	multi-region ^a^	12/2015 to	NA	19	85	Glecaprevir-Pibrentasvir
(2017) [25]		03/2016		(eGFR < 45 mL)		
Kawakami et al.	Japan	12/2014 to	3	NA	18	Daclatasvir (DCV) plus
(2016) [26]		01/2016	(eGFR ≥ 60 mL)			Asunaprevir (ASV)
Kondo et al.	Japan	09/2014 to	194	55	NA	DCV and ASV
(2017) [27]		09/2015	(eGFR ≥ 60 mL)	(eGFR < 60 mL)		
Morisawa et al.	Japan	09/2014 to	77	29	NA	DCV plus ASV
(2016) [28]		05/2015	(eGFR ≥ 60 mL)	(eGFR < 60 mL)		
Nakamura et al.	Japan	09/2014 to	126	21	NA	DCV plus ASV
(2017) [29]		08/2015	(eGFR ≥ 50 mL)	(eGFR < 50 mL)		
Puenpatom et al.	USA	11/2013 to	3202	236	NA	Sofosbuvir-based
(2017) [30]		06/2015	(eGFR ≥ 90 mL)	(eGFR < 90 mL)		regimens (SOF)
Roth et al.	multi-region ^b^	03/2014 to	NA	29	87	Grazoprevir plus
(2015) [19]		11/2014		(eGFR < 45 mL)		Elbasvir
Saxena et al.	North America	03/2015	1716	73	NA	SOF-based regimens
(2016) [31]	and Europe		(eGFR ≥ 60 mL)	(eGFR < 60 mL)		
Shin et al.	USA	12/2013 to	21	7	NA	SOF-based regimens
(2017) [32]		09/2015	(eGFR ≥ 45 mL)	(eGFR < 45 mL)		
Sho et al.	Japan	07/2014 to	191 (eGFR ≥ 60 mL)	40 (eGFR < 60 mL)	NA	SOF and ribavirin
(2018) [33]		05/2017	224 (eGFR ≥ 45 mL)	7 (eGFR < 45 mL)		
Sise et al.	USA	11/2013 to	74	24	NA	SOF-based therapy
(2017) [34]		12/2014	(eGFR ≥ 60 mL)	(eGFR < 60 mL)		
Suda et al.	Japan	07/2014 to	159 (eGFR ≥ 60 mL)	95 (eGFR < 60 mL)	NA	DCV and ASV
(2017) [35]		11/2016	298 (eGFR ≥ 45 mL)	24 (eGFR < 45 mL)		

^a^ Australia, Belgium, Canada, France, Greece, Italy, New Zealand, the United Kingdom, and the United States; ^b^ USA, Argentina, Australia, Canada, Estonia, France, Israel, South Korea, Lithuania, Netherlands, Spain, and Sweden; NA, not available; eGFR, estimated Glomerular filtration rate.

**Table:** (B)

	Age			Sex (Male)			
Location	None to Early	Advanced	Dialysis	None to Early	Advanced	Dialysis	Relevant Outcomes
Arai et al.	Overall: 67	(27–89)	NA	Overall: 117	(50%)	NA	RVR, SVR 12, VRET, rash (eruption),
(2018) [24]							ALT
Gane et al.	NA	Overall: 57	(28–83)	NA	Overall: 79	(76%)	SVR 12, adverse event
(2017) [25]							No comparison between groups
Kawakami et al.	80	NA	68	0	NA	14	ALT, diarrhea, fever, headache
(2016) [26]	(62–81)		(47–82)	(0%)		(78%)	
Kondo et al.	Overall: 71	(25–87)	NA	Overall: 105	(42%)	NA	RVR, SVR 12, VRET, rash (eruption),
(2017) [27]							ALT, renal disorder, discontinuation
Morisawa et al.	72.3 ± 7	74.9 ± 8	NA	28	13	NA	RVR, SVR 12, VRET, ALT,
(2016) [28]				(36%)	(45%)		discontinuation
Nakamura et al.	73 Me	78 Me	NA	52	4	NA	SVR 12, VRET, itching or rash (eruption),
(2017) [29]	(43–88)	(57–88)		(41%)	(21%)		ALT, discontinuation
Puenpatom et al.	58.76 ± 9.50	61.96 ± 7.74	NA	2013	167	NA	Rash (eruption), anemia, discontinuation
(2017) [30]				(62.87%)	(70.76%)		
Roth et al.	NA	NA	NA	NA	NA	NA	SVR 12
(2015) [19]							
Saxena et al.	*n* = 271(16%)	*n* = 17	NA	1107	33	NA	SVR 12, renal disorder, anemia
(2016) [31]	age ≥ 65	age ≥ 65		(65%)	(45%)		discontinuation
Shin et al.	61	62.9	NA	19	5	NA	SVR 12, rash (eruption), anemia,
(2017) [32]	(27–78)	(56–72)		(48%)	(71%)		discontinuation
Sho et al.	Overall: 62	(22–88)	NA	Overall: 106	(46%)	NA	RVR, SVR 12, VRET, ALT, renal disorder,
(2018) [33]							anemia, discontinuation
Sise et al.	61 ± 8	65 ± 10	NA	61	15	NA	SVR, adverse event
(2017) [34]				(82%)	(63%)		Without raw data.
Suda et al.	70.5 Me	70 Me	NA	103	10	NA	RVR, SVR 12, VRET, ALT, anemia,
(2017) [35]	(48–85)	(30–92)		(35%)	(42%)		renal disorder, discontinuation

RVR, rapid virologic response; SVR, sustained viral response; SVR 12, SVR at post-treatment week 12; VRET, virologic response at the end of treatment; ALT, alanine aminotransferase; NA, not available; Me, median.

**Table 2:** Summary of the network meta-analysis for ALT elevation and fever.

Comparisons			Events/Patients				Heterogeneity	
Group 1	Group 2	Studies	Group 1	Group 2	RR	95% CI	*I*-Square	*p*
Early CKD	Advanced CKD	4	10/162	30/750	0.73	(0.29–1.81)	29%	0.24
Early CKD	Dialysis	1	3/18	1/3	1.46	(0.18−12.03)	NA	NA
Advanced CKD	Dialysis	Indirect	NA	NA	2.00	(0.30−13.44)	NA	NA

**Table 3:** Summary of the meta-analysis for secondary outcomes (side effects and discontinuation).

Secondary		Events/Patients				Heterogeneity	
outcomes	Studies	None to Early	Advanced CKD	RR	95% CI	*I*-Square	*p*
Renal disorder	6	24/2585	14/246	0.14	(0.04−0.43)	35%	0.20
eGFR ≥ 60 vs. <60	5	20/2282	13/222	0.12	(0.04−0.43)	35%	0.22
eGFR ≥ 45 vs. <45	1	4/298	1/24	0.32	(0.04–2.77)	NA	NA
Anemia ^a^	5	328/5428	42/257	0.34	(0.20−0.57)	47%	0.11
eGFR ≥ 90 vs. <90	1	25/3202	6/113	0.15	(0.06–0.35)	NA	NA
eGFR ≥ 60 vs. <60	2	290/1907	32/113	0.49	(0.35–0.67)	0%	0.33
eGFR ≥ 45 vs. <45	2	13/319	4/31	0.32	(0.11–0.96)	0%	0.98
Eruption	5	71/3724	10/250	0.74	(0.14–3.28)	75%	>0.01
eGFR ≥ 90 vs. <90	1	36/3202	7/113	0.18	(0.08–0.40)	NA	NA
eGFR ≥ 60 vs. <60	2	5/375	1/109	0.84	(0.09–8.18)	23%	0.25
eGFR ≥ 50 vs. <50	1	29/126	2/21	2.42	(0.62–9.38)	NA	NA
eGFR ≥ 45 vs. <45	1	1/21	0/7	1.09	(0.05–24.13)	NA	NA
Discontinuation	8	439/5858	48/329	0.41	(0.30–0.56)	3%	0.40
eGFR ≥ 90 vs. <90	1	324/3202	31/113	0.37	(0.27–0.51)	NA	NA
eGFR ≥ 60 vs. <60	4	88/2178	15/197	0.50	(0.28–0.89)	0%	0.60
eGFR ≥ 50 vs. <50	1	1/126	1/21	0.17	(0.01–2.56)	NA	NA
eGFR ≥ 45 vs. <45	2	26/319	1/31	2.09	(0.30−14.77)	NA	NA

RR, Risk ratio; CKD, chronic kidney disease; ^a^ Hg < 10–8 g/dL; NA, not available; eGFR, estimated Glomerular filtration rate.

## Supplementary Materials

The following are available online at http://www.mdpi.com/2077-0383/7/10/314/s1, Table S1: Database and search strategy, Table S2: Risk of bias, Figure S1: Forest plot of SVR 12., Figure S2: Doi plot of SVR 12., Figure S3: Forest plot for SVR 12 in random-effects model., Figure S4: Forest plot for SVR 12 in fixed-effect model, Figure S5: Forest plots of rapid virologic response in fixed-effect model, Figure S6: Forest plots of virologic response at the end of treatment in fixed-effect model, Figure S7: Forest plot of paired comparisons for alanine aminotransferase elevation (3.0-5.0ULN), Figure S8: Forest plot of renal disorder in random-effects model., Figure S9: Forest plot of renal disorder in fixed-effect model, Figure S10: Forest plot of anemia in random-effects model., Figure S11: Forest plot of anemia in fixed-effect model, Figure S12: Forest plot of eruption in random-effects model, Figure S13: Forest plot of eruption in fixed-effect model, Figure S14: Forest plot of discontinuation in random-effects model, Figure S15: Forest plot of discontinuation in fixed-effect model, Figure S16: Funnel plot of discontinuation, Supplemental File 1 Results of publication bias of overall discontinuations.
