# Supplementary material for: Association of Renal Function and Direct-Acting Antiviral Agents for HCV: A Network Meta-Analysis
Source: J Clin Med. 2018 Sep 29;7(10):314. doi: 10.3390/jcm7100314 (PMC6210726; doi:10.3390/jcm7100314)

Association of Renal Function and Direct-Acting Antiviral Agents for HCV:

A Network Meta-Analysis

(Supplementary figures)

Index of Supplementary Figure

Supplementary Figure 1. Forest plot of SVR 12

Supplementary Figure 2. Doi plot of SVR12

Supplementary Figure 3. Forest plot for SVR 12 in random-effects model

Supplementary Figure 4. Forest plot for SVR 12 in fixed-effect model

Supplementary Figure 5. Forest plots of rapid virologic response in fixed-effect model

Supplementary Figure 6. Forest plots of virologic response at the end of treatment in fixed-effect model

Supplementary Figure 7. Forest plot of paired comparisons for alanine aminotransferase elevation

Supplementary Figure 8. Forest plot of renal disorder in random-effects model

Supplementary Figure 9. Forest plot of renal disorder in fixed-effect model

Supplementary Figure 10. Forest plot of anemia in random-effects model

Supplementary Figure 11. Forest plot of anemia in fixed-effect model

Supplementary Figure 12. Forest plot of eruption in random-effects model

Supplementary Figure 13. Forest plot of eruption in fixed-effect model

Supplementary Figure 14. Forest plot of discontinuation in random-effects model

Supplementary Figure 15. Forest plot of discontinuation in fixed-effect model

Supplementary Figure 16. Funnel plot of discontinuation

Supplementary Figure 1 to 3

Outcomes of sustained virologic response at post-treatment week 12 (SVR 12)

Supplementary Figure 1

Forest plot of SVR 12

SVR12 comparison: Others vs Early CKD

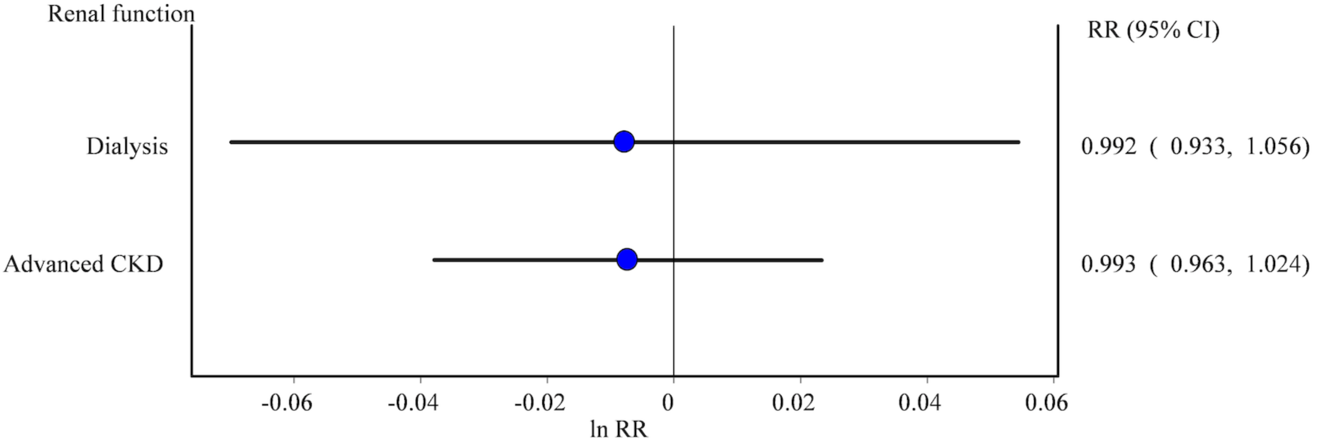

SVR12 comparison: Others vs Advanced CKD

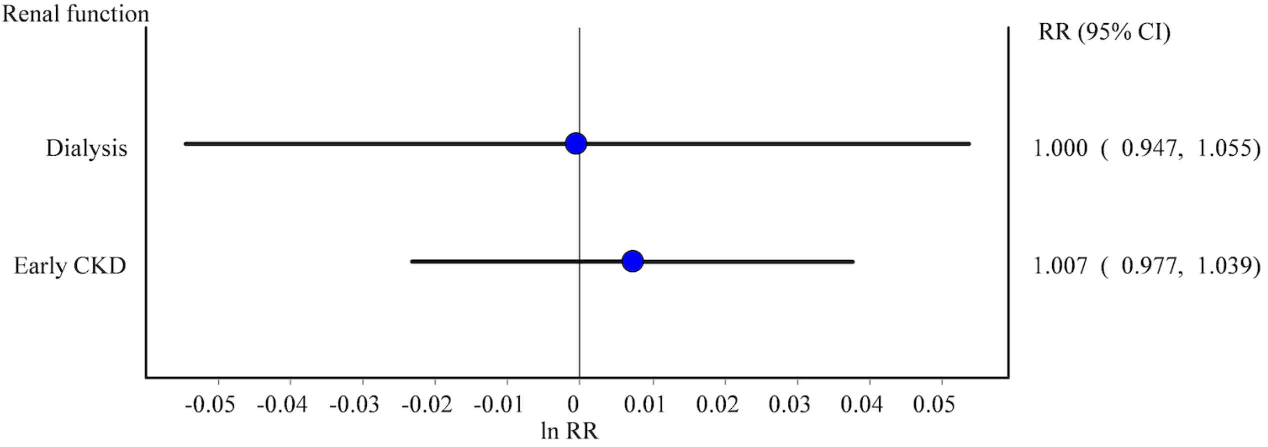

SVR12 comparison: Others vs Dialysis

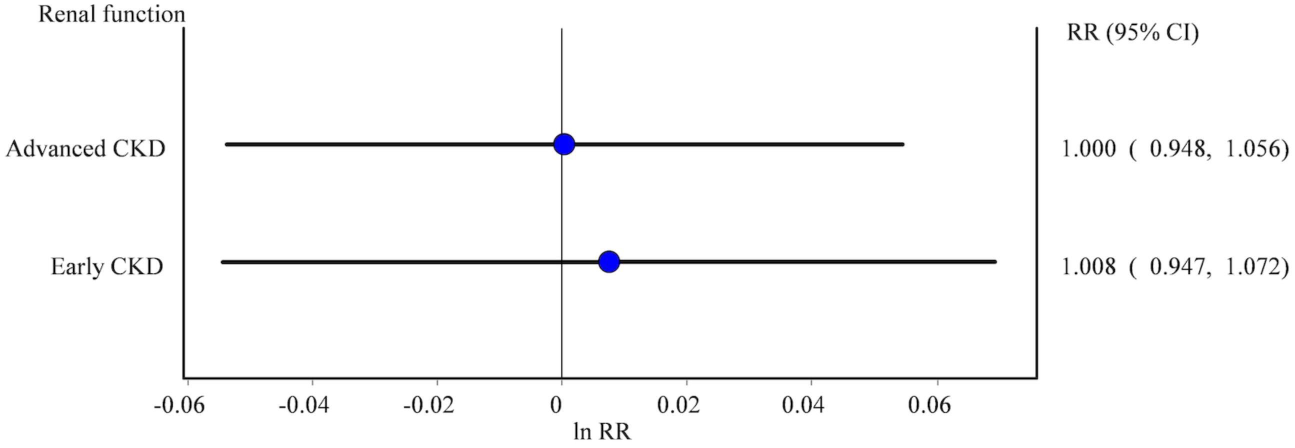

Supplementary Figure 2

Doi plot of SVR 12

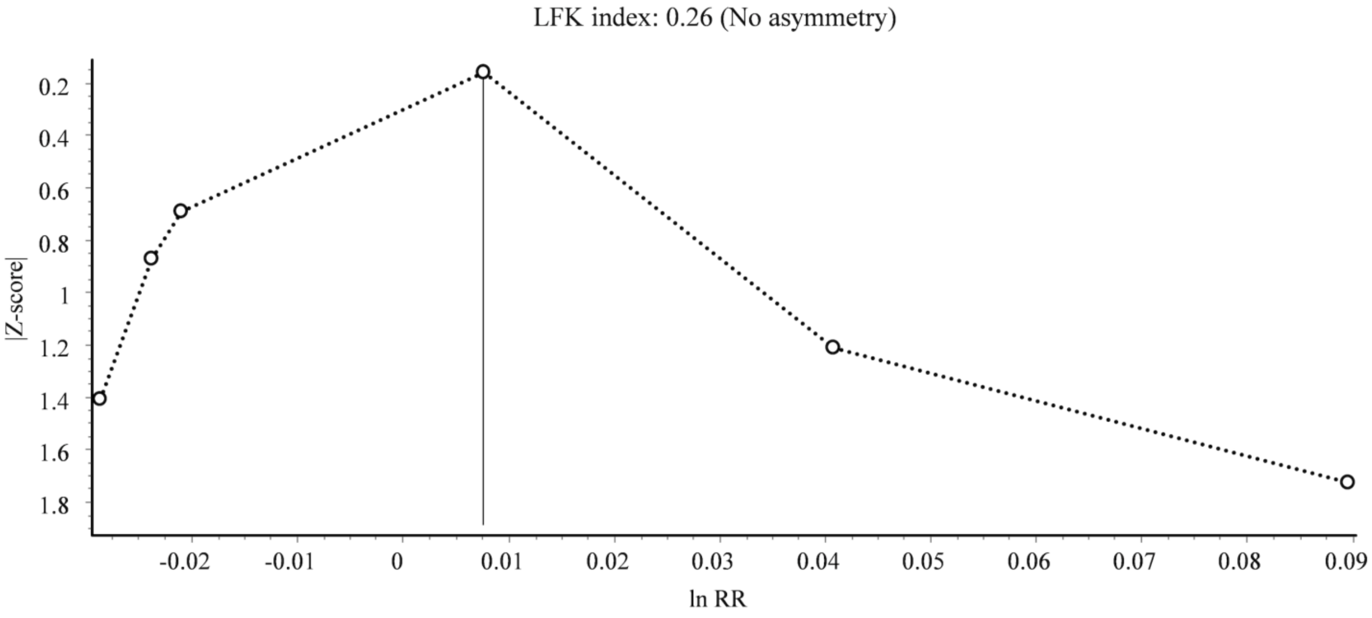

Supplementary Figure 3

Forest plot for SVR 12 in random-effects model

Study or Subgroup	Early CKD Events	Early CKD Total	Advanced CKD Events	Advanced CKD Total	Weight	Risk Ratio M-H, Random, 95% CI
**1.1.1 Sustained virologic response at post-treatment week 12 (eGFR ≥ 60 mL vs eGFR < 60 mL)**						
Kondo et al., 2017	169	194	46	55	13.6%	1.04 [0.92, 1.18]
Morisawa et al., 2016	70	77	27	29	14.4%	0.98 [0.86, 1.10]
Saxena et al., 2016	1220	1716	53	73	12.0%	0.98 [0.85, 1.13]
Sho et al., 2018	188	191	36	40	16.6%	1.09 [0.98, 1.21]
**Subtotal (95% CI)**		**2178**		**197**	**56.6%**	**1.03 [0.97, 1.10]**
Total events	1647		162			
Heterogeneity: Tau² = 0.00; Chi² = 2.60, df = 3 (P = 0.46); I² = 0%						
Test for overall effect: Z = 0.96 (P = 0.34)						
**1.1.2 Sustained virologic response at post-treatment week 12 (eGFR ≥ 50 mL vs eGFR < 50 mL)**						
Nakamura et al., 2017	119	126	20	21	16.7%	0.99 [0.89, 1.10]
**Subtotal (95% CI)**		**126**		**21**	**16.7%**	**0.99 [0.89, 1.10]**
Total events	119		20			
Heterogeneity: Not applicable						
Test for overall effect: Z = 0.16 (P = 0.88)						
**1.1.3 Sustained virologic response at post-treatment week 12 (eGFR ≥ 45 mL vs eGFR < 45 mL)**						
Shin et al., 2017	17	21	7	7	4.6%	0.85 [0.64, 1.12]
Suda et al., 2017	265	298	24	24	22.2%	0.91 [0.85, 0.97]
**Subtotal (95% CI)**		**319**		**31**	**26.7%**	**0.90 [0.84, 0.97]**
Total events	282		31			
Heterogeneity: Tau² = 0.00; Chi² = 0.27, df = 1 (P = 0.60); I² = 0%						
Test for overall effect: Z = 3.00 (P = 0.003)						
**Total (95% CI)**		**2623**		**249**	**100.0%**	**0.98 [0.92, 1.05]**
Total events	2048		213			
Heterogeneity: Tau² = 0.00; Chi² = 12.42, df = 6 (P = 0.05); I² = 52%						
Test for overall effect: Z = 0.50 (P = 0.62)						
Test for subgroup differences: Chi² = 8.32, df = 2 (P = 0.02), I² = 76.0%						

Risk Ratio
M-H, Random, 95% CI

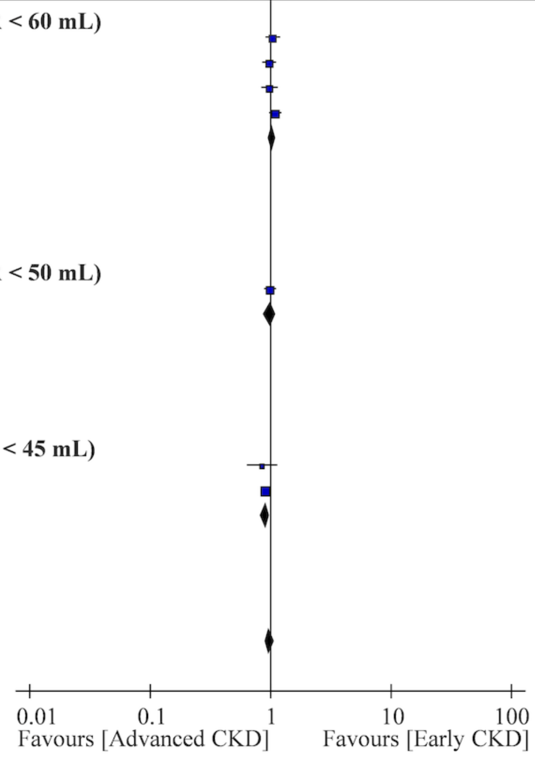

Supplementary Figure 4

Forest plot for SVR 12 in fixed-effect model

Study or Subgroup	Early CKD Events	Early CKD Total	Advanced CKD Events	Advanced CKD Total	Weight	Risk Ratio M-H, Fixed, 95% CI
**1.1.1 Sustained virologic response at post-treatment week 12 (eGFR ≥ 60 mL vs eGFR < 60 mL)**						
Kondo et al., 2017	169	194	46	55	19.8%	1.04 [0.92, 1.18]
Morisawa et al., 2016	70	77	27	29	10.8%	0.98 [0.86, 1.10]
Saxena et al., 2016	1220	1716	53	73	28.0%	0.98 [0.85, 1.13]
Sho et al., 2018	188	191	36	40	16.4%	1.09 [0.98, 1.21]
**Subtotal (95% CI)**		**2178**		**197**	**75.0%**	**1.02 [0.95, 1.09]**
Total events	1647		162			
Heterogeneity: Chi² = 2.60, df = 3 (P = 0.46); I² = 0%						
Test for overall effect: Z = 0.57 (P = 0.57)						
**1.1.2 Sustained virologic response at post-treatment week 12 (eGFR ≥ 50 mL vs eGFR < 50 mL)**						
Nakamura et al., 2017	119	126	20	21	9.5%	0.99 [0.89, 1.10]
**Subtotal (95% CI)**		**126**		**21**	**9.5%**	**0.99 [0.89, 1.10]**
Total events	119		20			
Heterogeneity: Not applicable						
Test for overall effect: Z = 0.16 (P = 0.88)						
**1.1.3 Sustained virologic response at post-treatment week 12 (eGFR ≥ 45 mL vs eGFR < 45 mL)**						
Shin et al., 2017	17	21	7	7	3.0%	0.85 [0.64, 1.12]
Suda et al., 2017	265	298	24	24	12.5%	0.91 [0.85, 0.97]
**Subtotal (95% CI)**		**319**		**31**	**15.5%**	**0.89 [0.83, 0.97]**
Total events	282		31			
Heterogeneity: Chi² = 0.27, df = 1 (P = 0.60); I² = 0%						
Test for overall effect: Z = 2.83 (P = 0.005)						
**Total (95% CI)**		**2623**		**249**	**100.0%**	**1.00 [0.94, 1.05]**
Total events	2048		213			
Heterogeneity: Chi² = 12.42, df = 6 (P = 0.05); I² = 52%						
Test for overall effect: Z = 0.07 (P = 0.95)						
Test for subgroup differences: Chi² = 6.43, df = 2 (P = 0.04), I² = 68.9%						

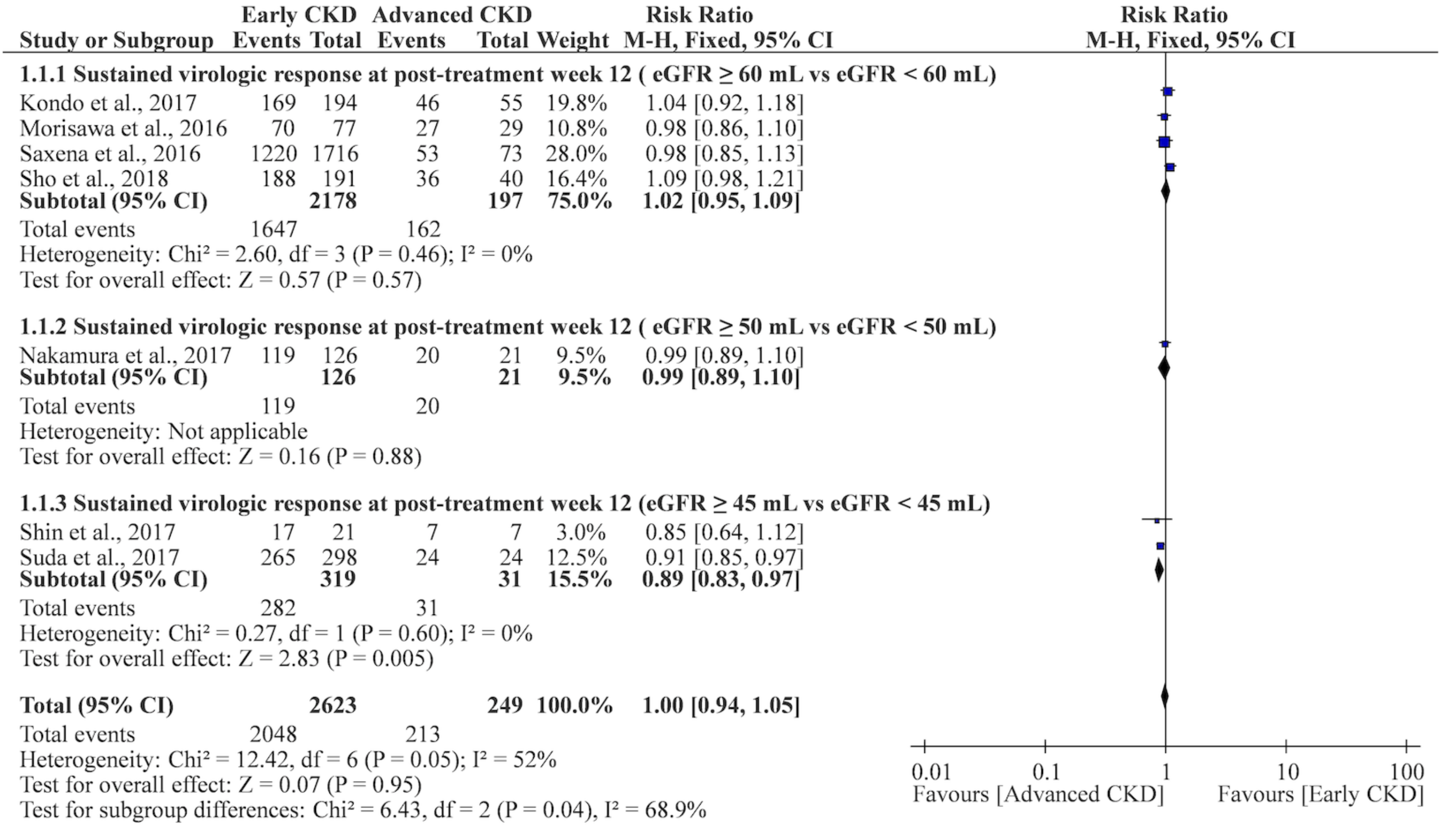

Supplementary Figure 5 to 6

Effectiveness outcomes in fix-effect model

Supplementary Figure 4

Forest plots of rapid virologic response in fixed-effect model

Study or Subgroup	Early CKD Events	Early CKD Total	Advanced CKD Events	Advanced CKD Total	Weight	Risk Ratio M-H, Fixed, 95% CI
**1.2.1 Rapid virological response (eGFR ≥ 60 mL vs eGFR < 60 mL)**						
Arai et al., 2018	140	181	45	54	25.9%	0.93 [0.80, 1.07]
Kondo et al. 2017	149	194	42	55	24.5%	1.01 [0.85, 1.19]
Morisawa et al. 2016	64	77	26	29	14.1%	0.93 [0.79, 1.09]
Sho et al., 2018	157	191	34	40	21.0%	0.97 [0.84, 1.12]
**Subtotal (95% CI)**		**643**		**178**	**85.5%**	**0.96 [0.89, 1.04]**
Total events	510		147			
Heterogeneity: Chi² = 0.71, df = 3 (P = 0.87); I² = 0%						
Test for overall effect: Z = 1.03 (P = 0.30)						
**1.2.2 Rapid virological response (eGFR ≥ 45 mL vs eGFR < 45 mL)**						
Suda et al. 2017	267	298	21	24	14.5%	1.02 [0.88, 1.20]
**Subtotal (95% CI)**		**298**		**24**	**14.5%**	**1.02 [0.88, 1.20]**
Total events	267		21			
Heterogeneity: Not applicable						
Test for overall effect: Z = 0.30 (P = 0.77)						
**Total (95% CI)**		**941**		**202**	**100.0%**	**0.97 [0.90, 1.04]**
Total events	777		168			
Heterogeneity: Chi² = 1.32, df = 4 (P = 0.86); I² = 0%						
Test for overall effect: Z = 0.87 (P = 0.38)						
Test for subgroup differences: Chi² = 0.53, df = 1 (P = 0.47), I² = 0%						

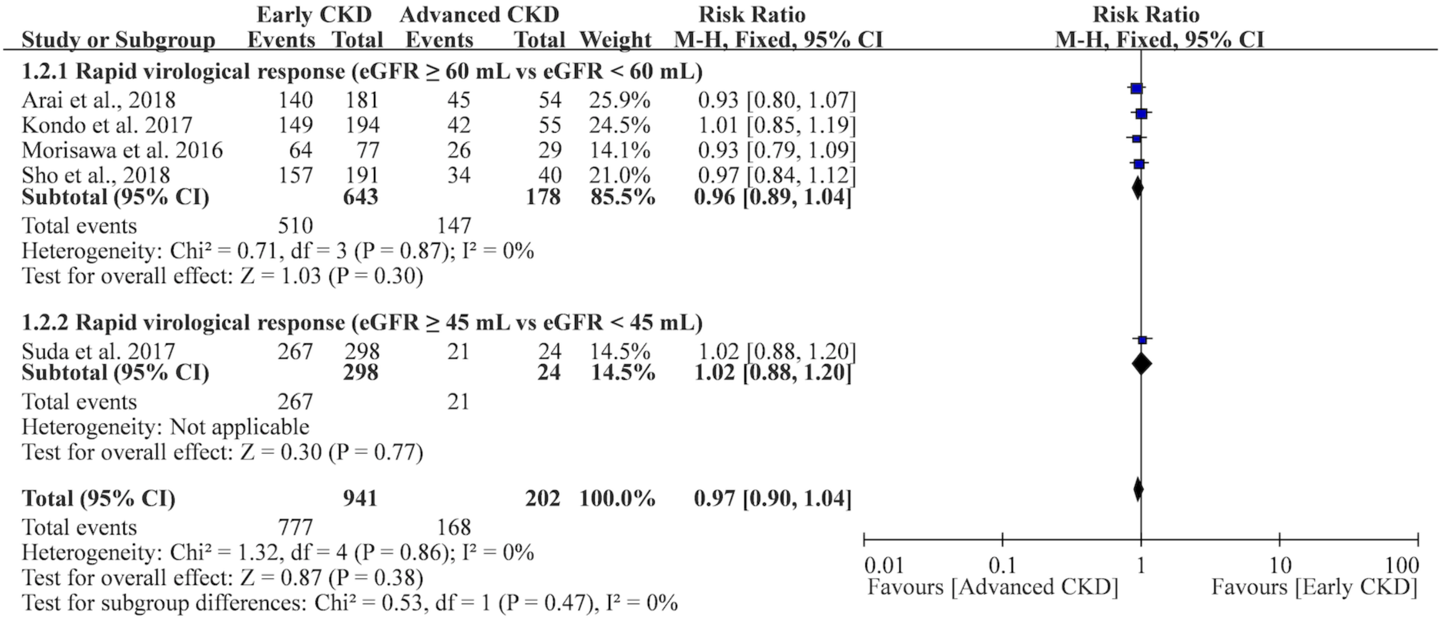

Supplementary Figure 5

Forest plots of virologic response at the end of treatment in fixed-effect model

Study or Subgroup	Early CKD Events	Early CKD Total	Advanced CKD Events	Advanced CKD Total	Weight	Risk Ratio M-H, Fixed, 95% CI
**1.3.1 Virus response at end of treatment (eGFR ≥ 60 mL vs eGFR < 60 mL)**						
Arai et al., 2018	179	181	53	54	23.6%	1.01 [0.97, 1.05]
Kondo et al., 2017	179	194	48	55	21.7%	1.06 [0.95, 1.18]
Morisawa et al., 2016	69	77	29	29	12.3%	0.91 [0.83, 0.99]
Sho et al., 2018	190	191	40	40	19.3%	1.00 [0.97, 1.04]
**Subtotal (95% CI)**		**643**		**178**	**77.0%**	**1.00 [0.97, 1.04]**
Total events	617		170			
Heterogeneity: Chi² = 5.85, df = 3 (P = 0.12); I² = 49%						
Test for overall effect: Z = 0.23 (P = 0.81)						
**1.3.2 Virus response at end of treatment (eGFR ≥ 50 mL vs eGFR < 50 mL)**						
Nakamura et al., 2017	122	126	20	21	9.9%	1.02 [0.92, 1.12]
**Subtotal (95% CI)**		**126**		**21**	**9.9%**	**1.02 [0.92, 1.12]**
Total events	122		20			
Heterogeneity: Not applicable						
Test for overall effect: Z = 0.32 (P = 0.75)						
**1.3.3 Virus response at end of treatment (eGFR ≥ 45 mL vs eGFR < 45 mL)**						
Suda et al., 2017	282	298	24	24	13.1%	0.96 [0.91, 1.03]
**Subtotal (95% CI)**		**298**		**24**	**13.1%**	**0.96 [0.91, 1.03]**
Total events	282		24			
Heterogeneity: Not applicable						
Test for overall effect: Z = 1.15 (P = 0.25)						
**Total (95% CI)**		**1067**		**223**	**100.0%**	**1.00 [0.97, 1.03]**
Total events	1021		214			
Heterogeneity: Chi² = 7.21, df = 5 (P = 0.21); I² = 31%						
Test for overall effect: Z = 0.03 (P = 0.98)						
Test for subgroup differences: Chi² = 1.40, df = 2 (P = 0.50), I² = 0%						

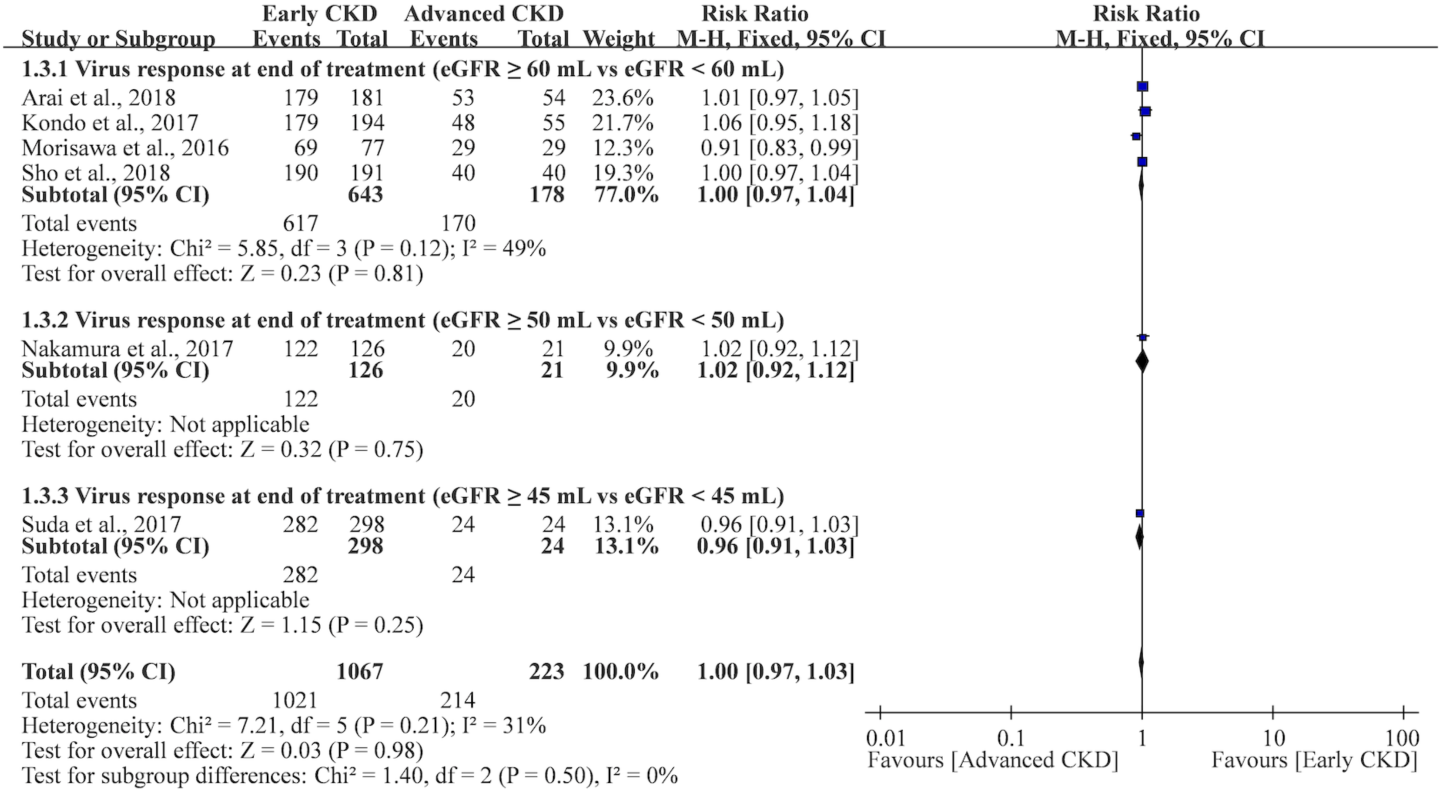

Supplementary Figure 7
Outcome of alanine aminotransferase elevation (ALT)

Supplementary Figure 7

Forest plot of paired comparisons for alanine aminotransferase elevation

ALT elevation(>3.0-5.0ULN) comparison: Others vs Early CKD

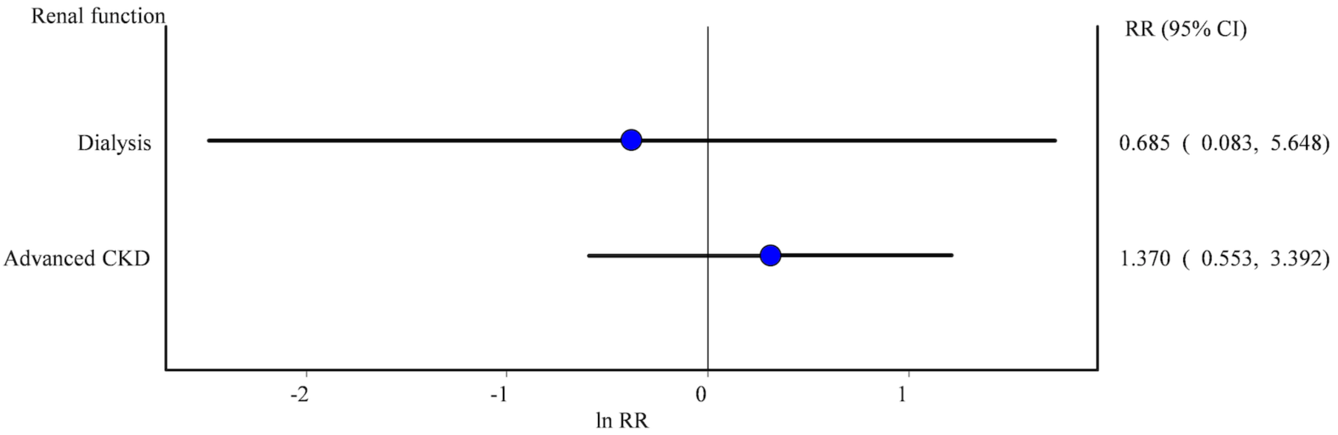

ALT elevation(>3.0-5.0ULN) comparison: Others vs Advanced CKD

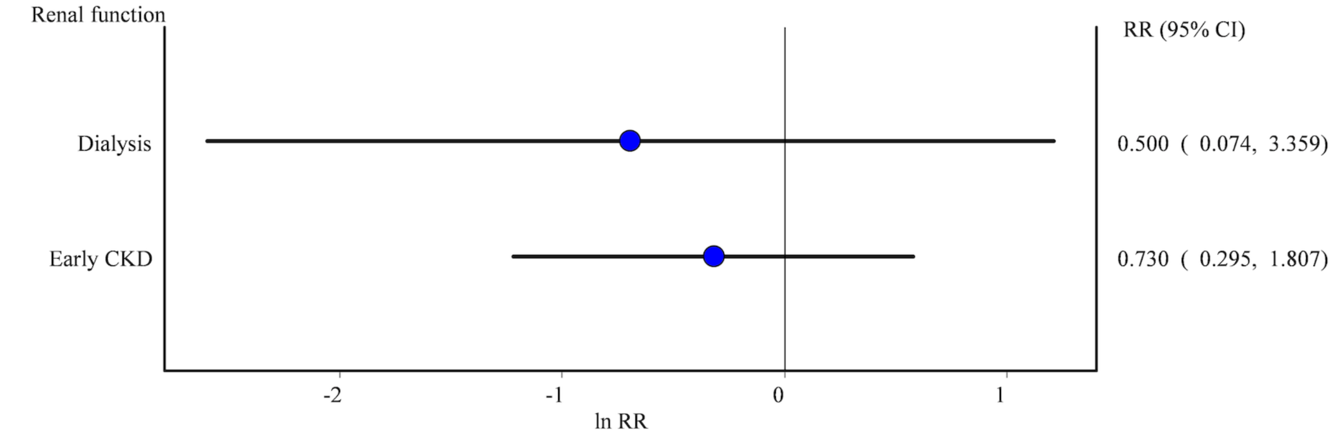

ALT elevation(>3.0-5.0ULN) comparison: Others vs Dialysis

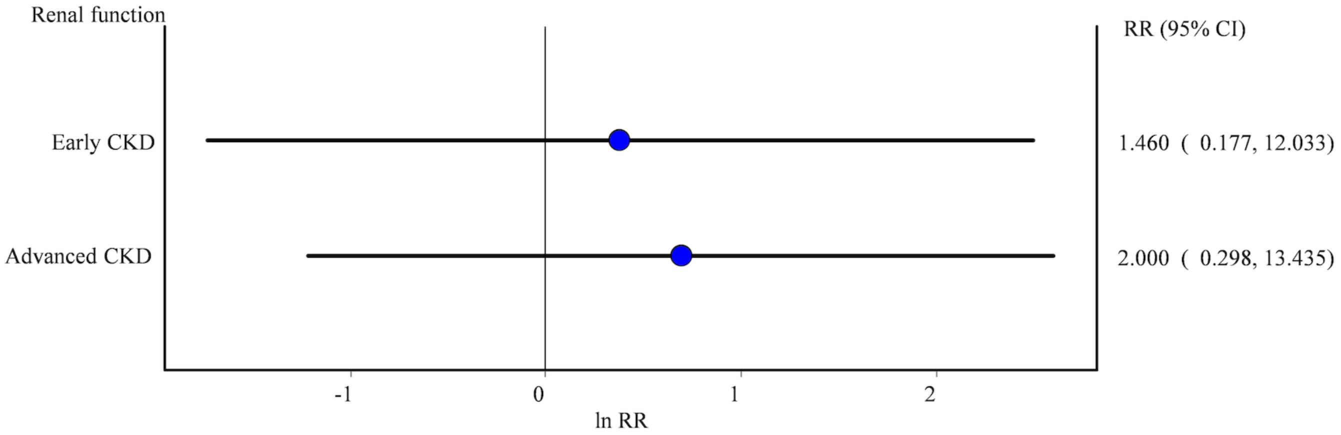

Supplementary Figure 8 to 9

Outcome of renal disorder

Supplementary Figure 8

Forest plot of renal disorder in random-effects model

Study or Subgroup	Early CKD Events	Early CKD Total	Advanced CKD Events	Advanced CKD Total	Weight	Risk Ratio M-H, Random, 95% CI
**2.1.1 Renal disorder (eGFR ≥ 60 mL vs eGFR < 60 mL)**						
Arai et al., 2018	1	181	0	54	10.5%	0.91 [0.04, 21.94]
Kondo et al., 2017	1	194	2	55	16.8%	0.14 [0.01, 1.53]
Saxena et al., 2016	18	1716	11	73	53.2%	0.07 [0.03, 0.14]
Sho et al., 2018	0	191	0	40		Not estimable
**Subtotal (95% CI)**		**2282**		**222**	**80.5%**	**0.12 [0.03, 0.43]**
Total events	20		13			
Heterogeneity: Tau² = 0.56; Chi² = 3.07, df = 2 (P = 0.22); I² = 35%						
Test for overall effect: Z = 3.23 (P = 0.001)						
**2.1.2 Renal disorder (eGFR ≥ 45 mL vs eGFR < 45 mL)**						
Suda et al., 2017	4	298	1	24	19.5%	0.32 [0.04, 2.77]
**Subtotal (95% CI)**		**298**		**24**	**19.5%**	**0.32 [0.04, 2.77]**
Total events	4		1			
Heterogeneity: Not applicable						
Test for overall effect: Z = 1.03 (P = 0.30)						
**Total (95% CI)**		**2580**		**246**	**100.0%**	**0.14 [0.04, 0.43]**
Total events	24		14			
Heterogeneity: Tau² = 0.49; Chi² = 4.59, df = 3 (P = 0.20); I² = 35%						
Test for overall effect: Z = 3.44 (P = 0.0006)						
Test for subgroup differences: Chi² = 0.63, df = 1 (P = 0.43), I² = 0%						

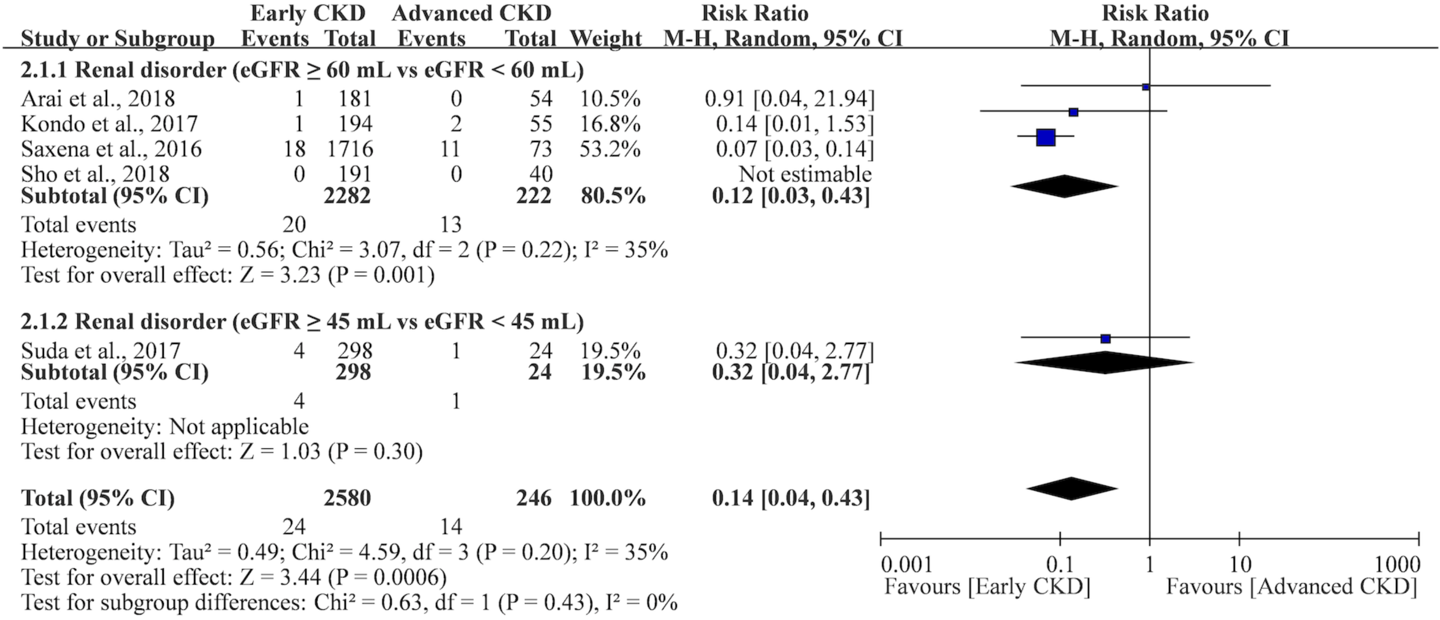

Supplementary Figure 9

Forest plot of renal disorder in fixed-effect model

Study or Subgroup	Early CKD Events	Early CKD Total	Advanced CKD Events	Advanced CKD Total	Weight	Peto Odds Ratio Peto, Fixed, 95% CI
**2.1.1 Renal disorder (eGFR ≥ 60 mL vs eGFR < 60 mL)**						
Arai et al., 2018	1	181	0	54	8.2%	3.66 [0.03, 386.53]
Kondo et al., 2017	1	194	2	55	23.9%	0.07 [0.00, 1.14]
Saxena et al., 2016	18	1716	11	73	52.0%	0.00 [0.00, 0.00]
Sho et al., 2018	0	191	0	40		Not estimable
**Subtotal (95% CI)**		**2282**		**222**	**84.1%**	**0.00 [0.00, 0.01]**
Total events	20		13			
Heterogeneity: Chi² = 23.98, df = 2 (P < 0.00001); I² = 92%						
Test for overall effect: Z = 8.13 (P < 0.00001)						
**2.1.2 Renal disorder (eGFR ≥ 45 mL vs eGFR < 45 mL)**						
Suda et al., 2017	4	298	1	24	15.9%	0.16 [0.01, 4.56]
**Subtotal (95% CI)**		**298**		**24**	**15.9%**	**0.16 [0.01, 4.56]**
Total events	4		1			
Heterogeneity: Not applicable						
Test for overall effect: Z = 1.07 (P = 0.28)						
**Total (95% CI)**		**2580**		**246**	**100.0%**	**0.00 [0.00, 0.02]**
Total events	24		14			
Heterogeneity: Chi² = 29.05, df = 3 (P < 0.00001); I² = 90%						
Test for overall effect: Z = 7.88 (P < 0.00001)						
Test for subgroup differences: Chi² = 5.07, df = 1 (P = 0.02), I² = 80.3%						

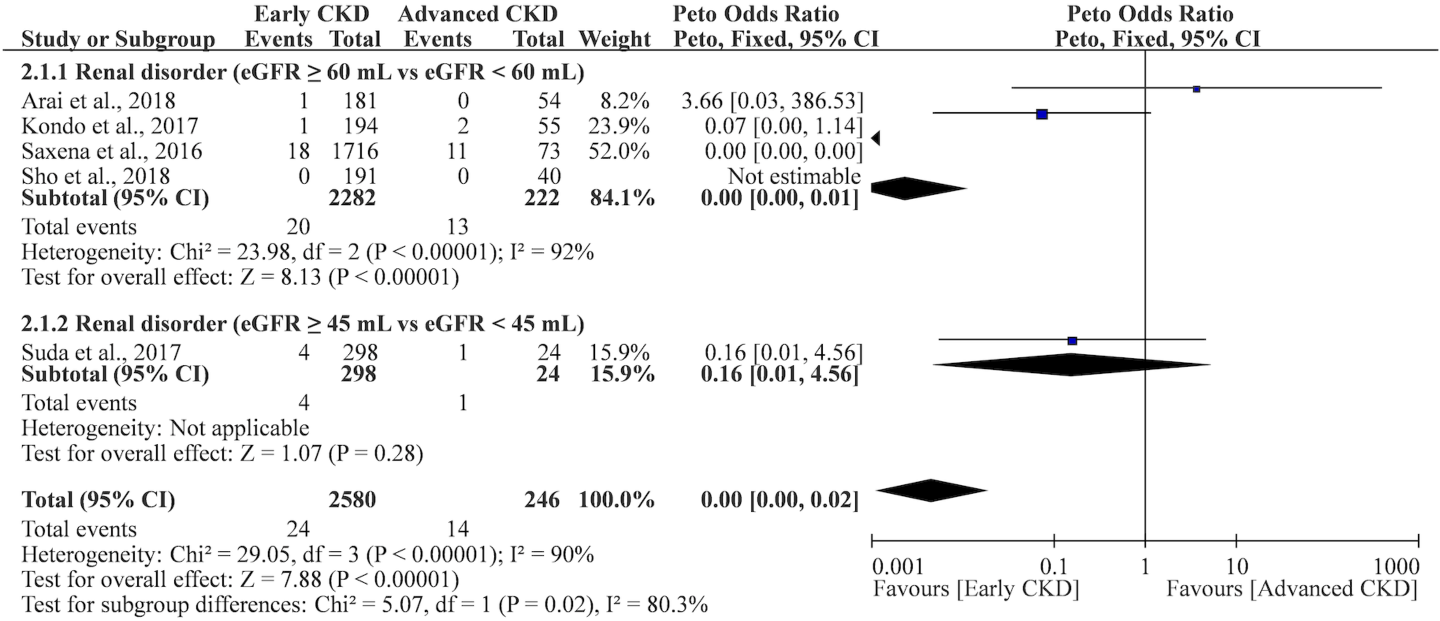

Supplementary Figure 10 to 11

Outcome of anemia

Supplementary Figure 10

Forest plot of anemia in random-effects model

Study or Subgroup	Early CKD Events	Early CKD Total	Advanced CKD Events	Advanced CKD Total	Weight	Risk Ratio M-H, Random, 95% CI
**2.2.1 Anemia (Hg<10-8 g/dL; eGFR ≥ 90 mL vs eGFR < 90 mL)**						
Puenpatom et al., 2017	25	3202	6	113	20.0%	0.15 [0.06, 0.35]
**Subtotal (95% CI)**		**3202**		**113**	**20.0%**	**0.15 [0.06, 0.35]**
Total events	25		6			
Heterogeneity: Not applicable						
Test for overall effect: Z = 4.31 (P < 0.0001)						
**2.2.2 Anemia (Hg<10-8 g/dL; eGFR ≥ 60 mL vs eGFR < 60 mL)**						
Saxena et al., 2016	273	1716	22	73	38.2%	0.53 [0.37, 0.76]
Sho et al., 2018	17	191	10	40	25.1%	0.36 [0.18, 0.72]
**Subtotal (95% CI)**		**1907**		**113**	**63.3%**	**0.49 [0.35, 0.67]**
Total events	290		32			
Heterogeneity: $Tau^2 = 0.00$; $Chi^2 = 0.95$, df = 1 (P = 0.33); $I^2 = 0\%$						
Test for overall effect: Z = 4.37 (P < 0.0001)						
**2.2.3 Anemia (Hg<10-8 g/dL; eGFR ≥ 45 mL vs eGFR < 45 mL)**						
Shin et al., 2017	1	21	1	7	3.5%	0.33 [0.02, 4.65]
Suda et al., 2017	12	298	3	24	13.2%	0.32 [0.10, 1.06]
**Subtotal (95% CI)**		**319**		**31**	**16.7%**	**0.32 [0.11, 0.96]**
Total events	13		4			
Heterogeneity: $Tau^2 = 0.00$; $Chi^2 = 0.00$, df = 1 (P = 0.98); $I^2 = 0\%$						
Test for overall effect: Z = 2.03 (P = 0.04)						
**Total (95% CI)**		**5428**		**257**	**100.0%**	**0.34 [0.20, 0.57]**
Total events	328		42			
Heterogeneity: $Tau^2 = 0.14$; $Chi^2 = 7.50$, df = 4 (P = 0.11); $I^2 = 47\%$						
Test for overall effect: Z = 4.11 (P < 0.0001)						
Test for subgroup differences: $Chi^2 = 6.54$, df = 2 (P = 0.04), $I^2 = 69.4\%$						

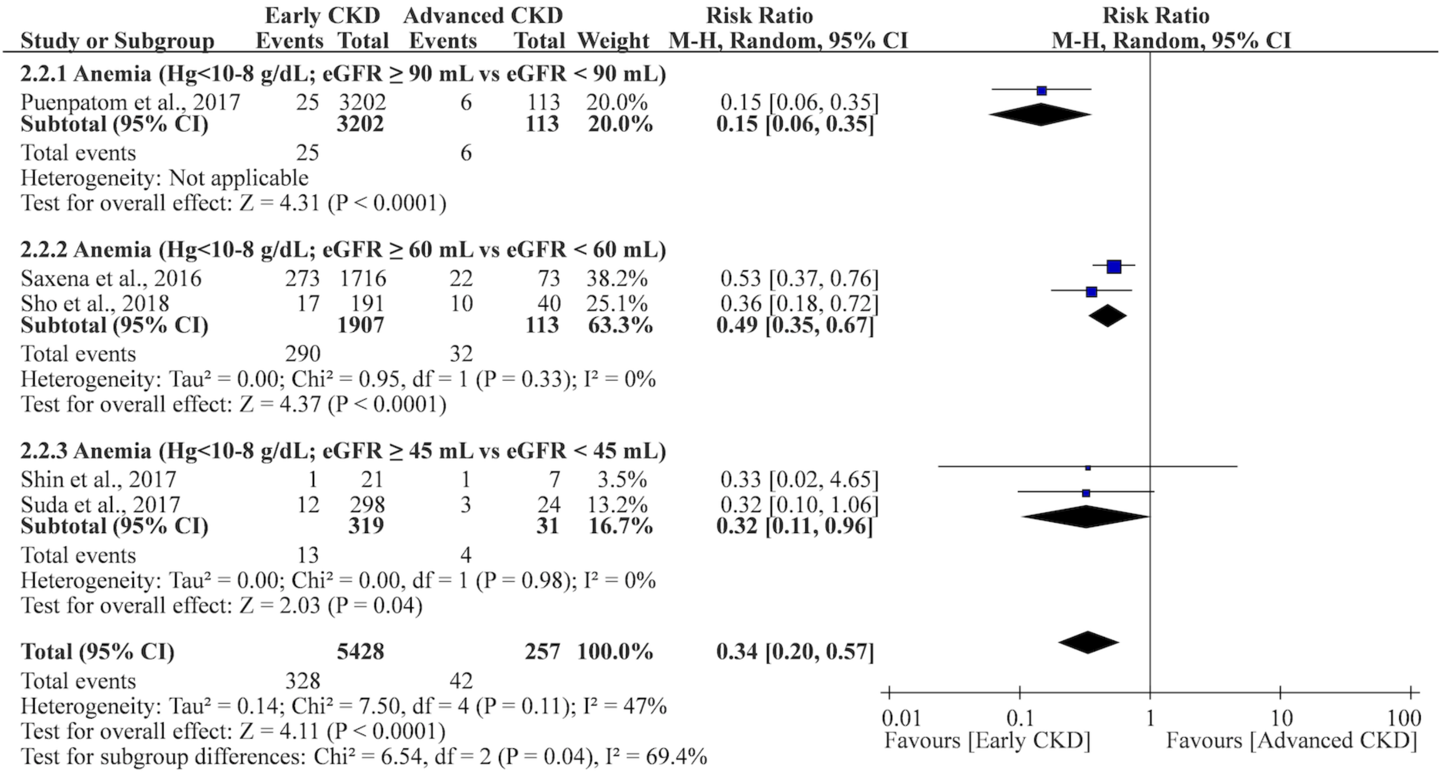

Supplementary Figure 11

Forest plot of anemia in fixed-effect model

Study or Subgroup	Early CKD Events	Early CKD Total	Advanced CKD Events	Advanced CKD Total	Weight	Risk Ratio M-H, Fixed, 95% CI
**2.2.1 Anemia (Hg<10-8 g/dL; eGFR ≥ 90 mL vs eGFR < 90 mL)**						
Puenpatom et al., 2017	25	3202	6	113	15.0%	0.15 [0.06, 0.35]
**Subtotal (95% CI)**		**3202**		**113**	**15.0%**	**0.15 [0.06, 0.35]**
Total events	25		6			
Heterogeneity: Not applicable						
Test for overall effect: Z = 4.31 (P < 0.0001)						
**2.2.2 Anemia (Hg<10-8 g/dL; eGFR ≥ 60 mL vs eGFR < 60 mL)**						
Saxena et al., 2016	273	1716	22	73	54.5%	0.53 [0.37, 0.76]
Sho et al., 2018	17	191	10	40	21.4%	0.36 [0.18, 0.72]
**Subtotal (95% CI)**		**1907**		**113**	**75.9%**	**0.48 [0.35, 0.66]**
Total events	290		32			
Heterogeneity: Chi² = 0.95, df = 1 (P = 0.33); I² = 0%						
Test for overall effect: Z = 4.44 (P < 0.00001)						
**2.2.3 Anemia (Hg<10-8 g/dL; eGFR ≥ 45 mL vs eGFR < 45 mL)**						
Shin et al., 2017	1	21	1	7	1.9%	0.33 [0.02, 4.65]
Suda et al., 2017	12	298	3	24	7.2%	0.32 [0.10, 1.06]
**Subtotal (95% CI)**		**319**		**31**	**9.1%**	**0.32 [0.11, 0.97]**
Total events	13		4			
Heterogeneity: Chi² = 0.00, df = 1 (P = 0.98); I² = 0%						
Test for overall effect: Z = 2.01 (P = 0.04)						
**Total (95% CI)**		**5428**		**257**	**100.0%**	**0.42 [0.31, 0.56]**
Total events	328		42			
Heterogeneity: Chi² = 7.50, df = 4 (P = 0.11); I² = 47%						
Test for overall effect: Z = 5.95 (P < 0.00001)						
Test for subgroup differences: Chi² = 6.39, df = 2 (P = 0.04), I² = 68.7%						

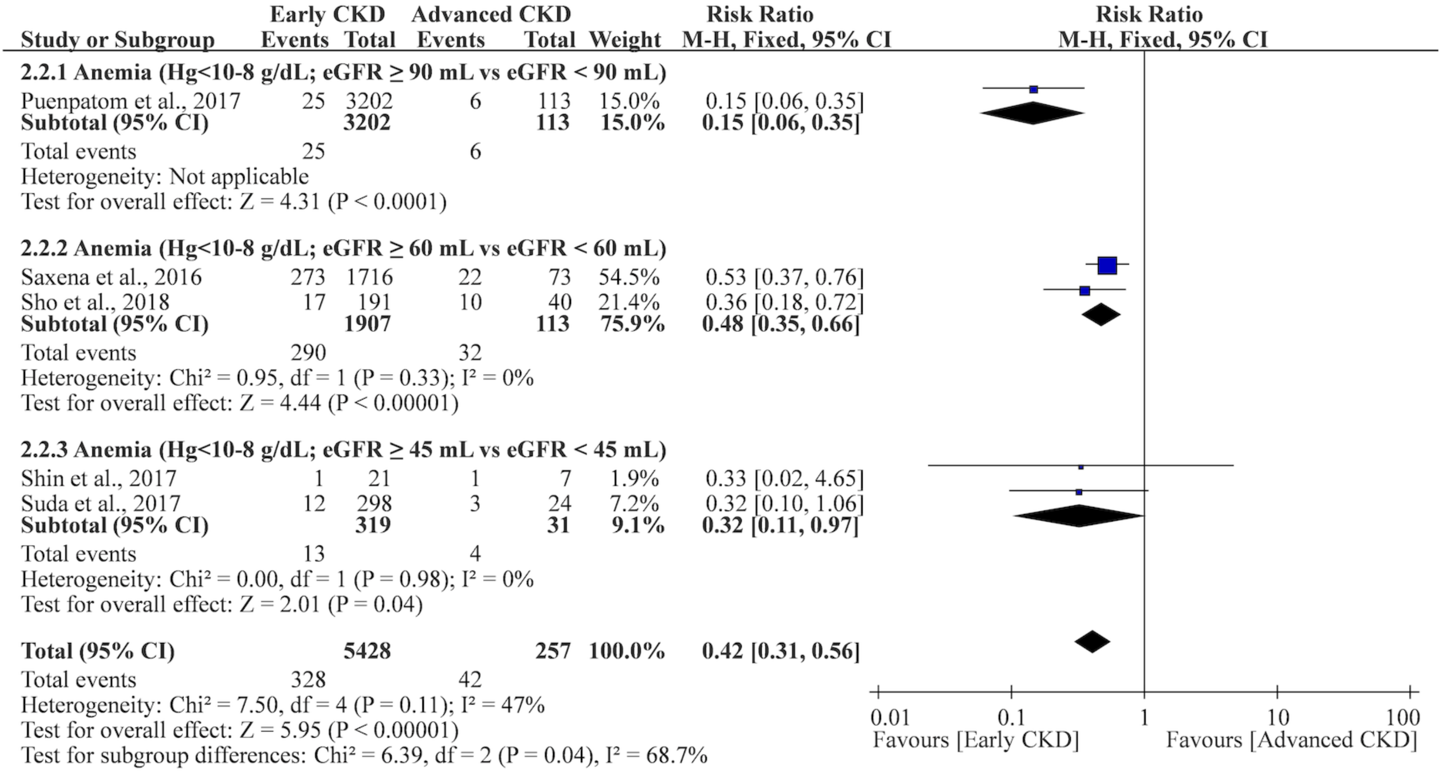

Supplementary Figure 12 to 13

Outcome of eruption

Supplementary Figure 12

Forest plot of eruption in random-effects model

Study or Subgroup	Early CKD Events	Early CKD Total	Advanced CKD Events	Advanced CKD Total	Weight	Risk Ratio M-H, Random, 95% CI
**2.3.1 Eruption (eGFR ≥ 90 mL vs eGFR < 90 mL)**						
Puenpatom et al., 2017	36	3202	7	113	28.4%	0.18 [0.08, 0.40]
**Subtotal (95% CI)**		**3202**		**113**	**28.4%**	**0.18 [0.08, 0.40]**
Total events	36		7			
Heterogeneity: Not applicable						
Test for overall effect: Z = 4.25 (P < 0.0001)						
**2.3.2 Eruption (eGFR ≥ 60 mL vs eGFR < 60 mL)**						
Arai et al., 2018	4	181	0	54	15.5%	2.72 [0.15, 49.73]
Kondo et al., 2017	1	194	1	55	16.3%	0.28 [0.02, 4.46]
**Subtotal (95% CI)**		**375**		**109**	**31.9%**	**0.84 [0.09, 8.18]**
Total events	5		1			
Heterogeneity: Tau² = 0.62; Chi² = 1.30, df = 1 (P = 0.25); I² = 23%						
Test for overall effect: Z = 0.15 (P = 0.88)						
**2.3.3 Eruption (eGFR ≥ 50 mL vs eGFR < 50 mL)**						
Nakamura et al., 2017	29	126	2	21	25.1%	2.42 [0.62, 9.38]
**Subtotal (95% CI)**		**126**		**21**	**25.1%**	**2.42 [0.62, 9.38]**
Total events	29		2			
Heterogeneity: Not applicable						
Test for overall effect: Z = 1.28 (P = 0.20)						
**2.3.4 Eruption (eGFR ≥ 45 mL vs eGFR < 45 mL)**						
Shin et al., 2017	1	21	0	7	14.6%	1.09 [0.05, 24.13]
**Subtotal (95% CI)**		**21**		**7**	**14.6%**	**1.09 [0.05, 24.13]**
Total events	1		0			
Heterogeneity: Not applicable						
Test for overall effect: Z = 0.06 (P = 0.96)						
**Total (95% CI)**		**3724**		**250**	**100.0%**	**0.74 [0.14, 3.82]**
Total events	71		10			
Heterogeneity: Tau² = 2.31; Chi² = 15.87, df = 4 (P = 0.003); I² = 75%						
Test for overall effect: Z = 0.36 (P = 0.72)						
Test for subgroup differences: Chi² = 11.43, df = 3 (P = 0.010), I² = 73.7%						

Risk Ratio
M-H, Random, 95% CI

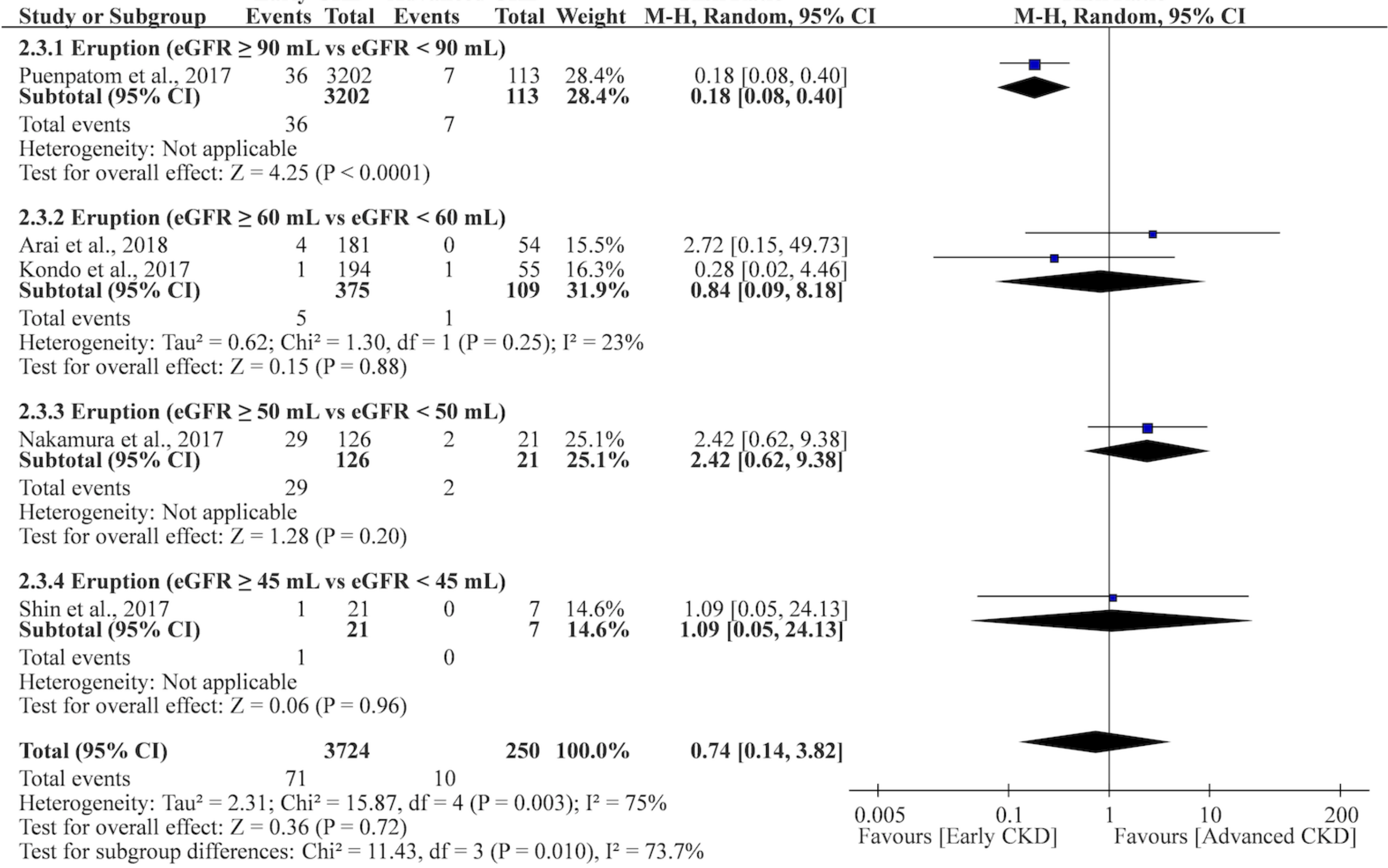

Supplementary Figure 13

Forest plot of eruption in fixed-effect model

Study or Subgroup	Early CKD Events	Early CKD Total	Advanced CKD Events	Advanced CKD Total	Weight	Peto Odds Ratio Peto, Fixed, 95% CI
**2.3.1 Eruption (eGFR ≥ 90 mL vs eGFR < 90 mL)**						
Puenpatom et al., 2017	36	3202	7	113	24.8%	0.02 [0.00, 0.10]
**Subtotal (95% CI)**		**3202**		**113**	**24.8%**	**0.02 [0.00, 0.10]**
Total events	36		7			
Heterogeneity: Not applicable						
Test for overall effect: Z = 4.68 (P < 0.00001)						
**2.3.2 Eruption (eGFR ≥ 60 mL vs eGFR < 60 mL)**						
Arai et al., 2018	4	181	0	54	12.4%	3.73 [0.36, 38.85]
Kondo et al., 2017	1	194	1	55	6.1%	0.20 [0.01, 5.58]
**Subtotal (95% CI)**		**375**		**109**	**18.5%**	**1.41 [0.21, 9.65]**
Total events	5		1			
Heterogeneity: $Chi^2 = 1.99$, df = 1 (P = 0.16); $I^2 = 50\%$						
Test for overall effect: Z = 0.35 (P = 0.72)						
**2.3.3 Eruption (eGFR ≥ 50 mL vs eGFR < 50 mL)**						
Nakamura et al., 2017	29	126	2	21	53.4%	2.24 [0.72, 6.92]
**Subtotal (95% CI)**		**126**		**21**	**53.4%**	**2.24 [0.72, 6.92]**
Total events	29		2			
Heterogeneity: Not applicable						
Test for overall effect: Z = 1.40 (P = 0.16)						
**2.3.4 Eruption (eGFR ≥ 45 mL vs eGFR < 45 mL)**						
Shin et al., 2017	1	21	0	7	3.3%	3.79 [0.04, 350.61]
**Subtotal (95% CI)**		**21**		**7**	**3.3%**	**3.79 [0.04, 350.61]**
Total events	1		0			
Heterogeneity: Not applicable						
Test for overall effect: Z = 0.58 (P = 0.56)						
**Total (95% CI)**		**3724**		**250**	**100.0%**	**0.64 [0.28, 1.47]**
Total events	71		10			
Heterogeneity: $Chi^2 = 25.21$, df = 4 (P < 0.0001); $I^2 = 84\%$						
Test for overall effect: Z = 1.05 (P = 0.29)						
Test for subgroup differences: $Chi^2 = 23.22$, df = 3 (P < 0.0001), $I^2 = 87.1\%$						

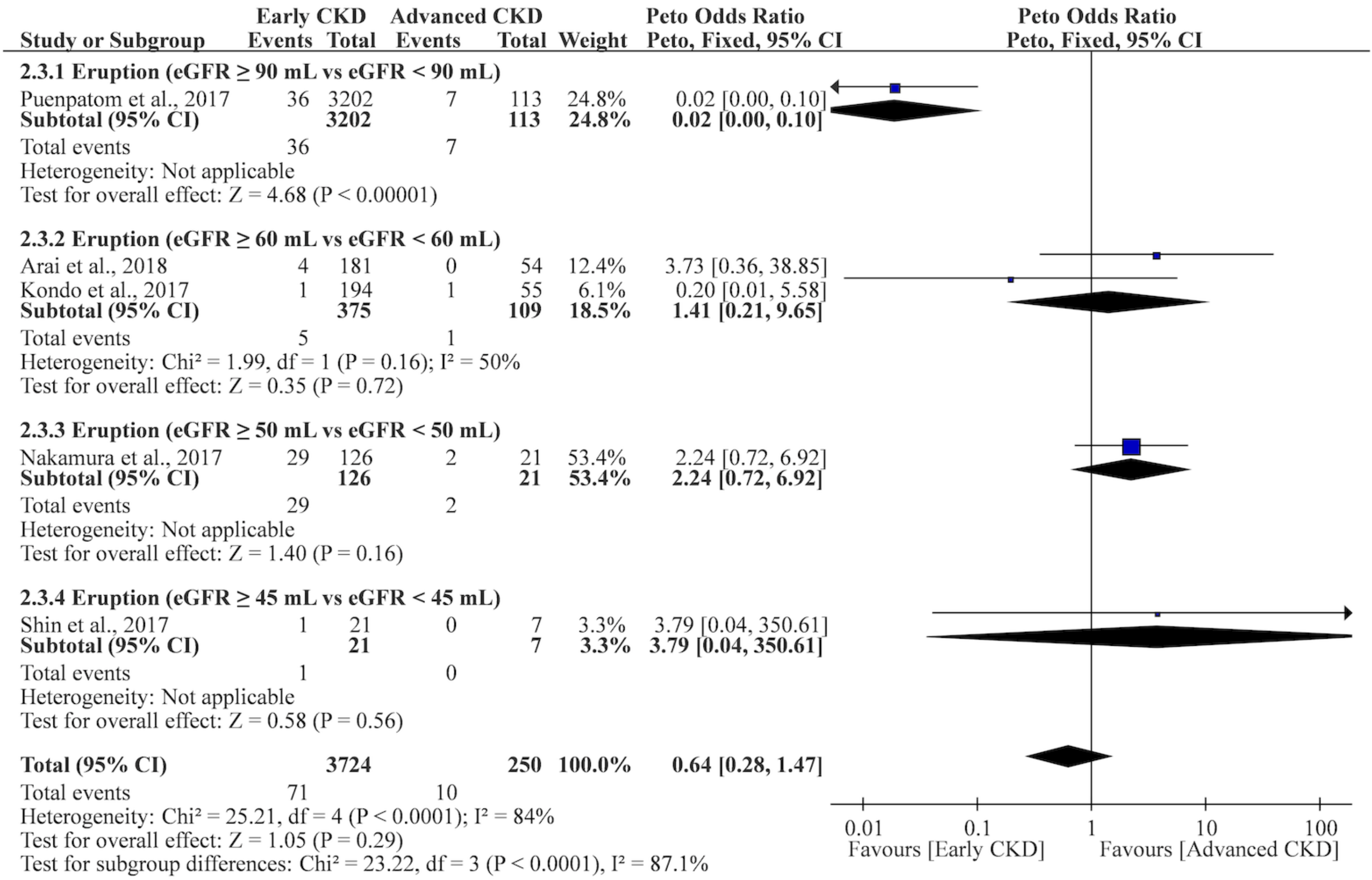

Supplementary Figure 14 to 16

Outcomes of discontinuation

Supplementary Figure 14

Forest plot of discontinuation in random-effects model

Study or Subgroup	Early CKD Events	Early CKD Total	Advanced CKD Events	Advanced CKD Total	Weight	Risk Ratio M-H, Random, 95% CI
**2.4.1 Overall discontinuation (eGFR ≥ 90 mL vs eGFR < 90 mL)**						
Puenpatom et al., 2017	324	3202	31	113	69.5%	0.37 [0.27, 0.51]
**Subtotal (95% CI)**		**3202**		**113**	**69.5%**	**0.37 [0.27, 0.51]**
Total events	324		31			
Heterogeneity: Not applicable						
Test for overall effect: Z = 6.16 (P < 0.00001)						
**2.4.2 Overall discontinuation (eGFR ≥ 60 mL vs eGFR < 60 mL)**						
Kondo et al., 2017	7	194	7	55	9.0%	0.28 [0.10, 0.77]
Morisawa et al., 2016	6	77	3	29	5.3%	0.75 [0.20, 2.82]
Saxena et al., 2016	74	1716	5	73	11.7%	0.63 [0.26, 1.51]
Sho et al., 2018	1	191	0	40	0.9%	0.64 [0.03, 15.45]
**Subtotal (95% CI)**		**2178**		**197**	**26.8%**	**0.50 [0.28, 0.89]**
Total events	88		15			
Heterogeneity: $Tau^2 = 0.00$; $Chi^2 = 1.89$, df = 3 (P = 0.60); $I^2 = 0\%$						
Test for overall effect: Z = 2.34 (P = 0.02)						
**2.4.3 Overall discontinuation (eGFR ≥ 50 mL vs eGFR < 50 mL)**						
Nakamura et al., 2017	1	126	1	21	1.2%	0.17 [0.01, 2.56]
**Subtotal (95% CI)**		**126**		**21**	**1.2%**	**0.17 [0.01, 2.56]**
Total events	1		1			
Heterogeneity: Not applicable						
Test for overall effect: Z = 1.28 (P = 0.20)						
**2.4.4 Overall discontinuation (eGFR ≥ 45 mL vs eGFR < 45 mL)**						
Shin et al., 2017	0	21	0	7		Not estimable
Suda et al., 2017	26	298	1	24	2.4%	2.09 [0.30, 14.77]
**Subtotal (95% CI)**		**319**		**31**	**2.4%**	**2.09 [0.30, 14.77]**
Total events	26		1			
Heterogeneity: Not applicable						
Test for overall effect: Z = 0.74 (P = 0.46)						
**Total (95% CI)**		**5825**		**362**	**100.0%**	**0.41 [0.30, 0.56]**
Total events	439		48			
Heterogeneity: $Tau^2 = 0.01$; $Chi^2 = 6.19$, df = 6 (P = 0.40); $I^2 = 3\%$						
Test for overall effect: Z = 5.67 (P < 0.00001)						
Test for subgroup differences: $Chi^2 = 3.96$, df = 3 (P = 0.27), $I^2 = 24.3\%$						

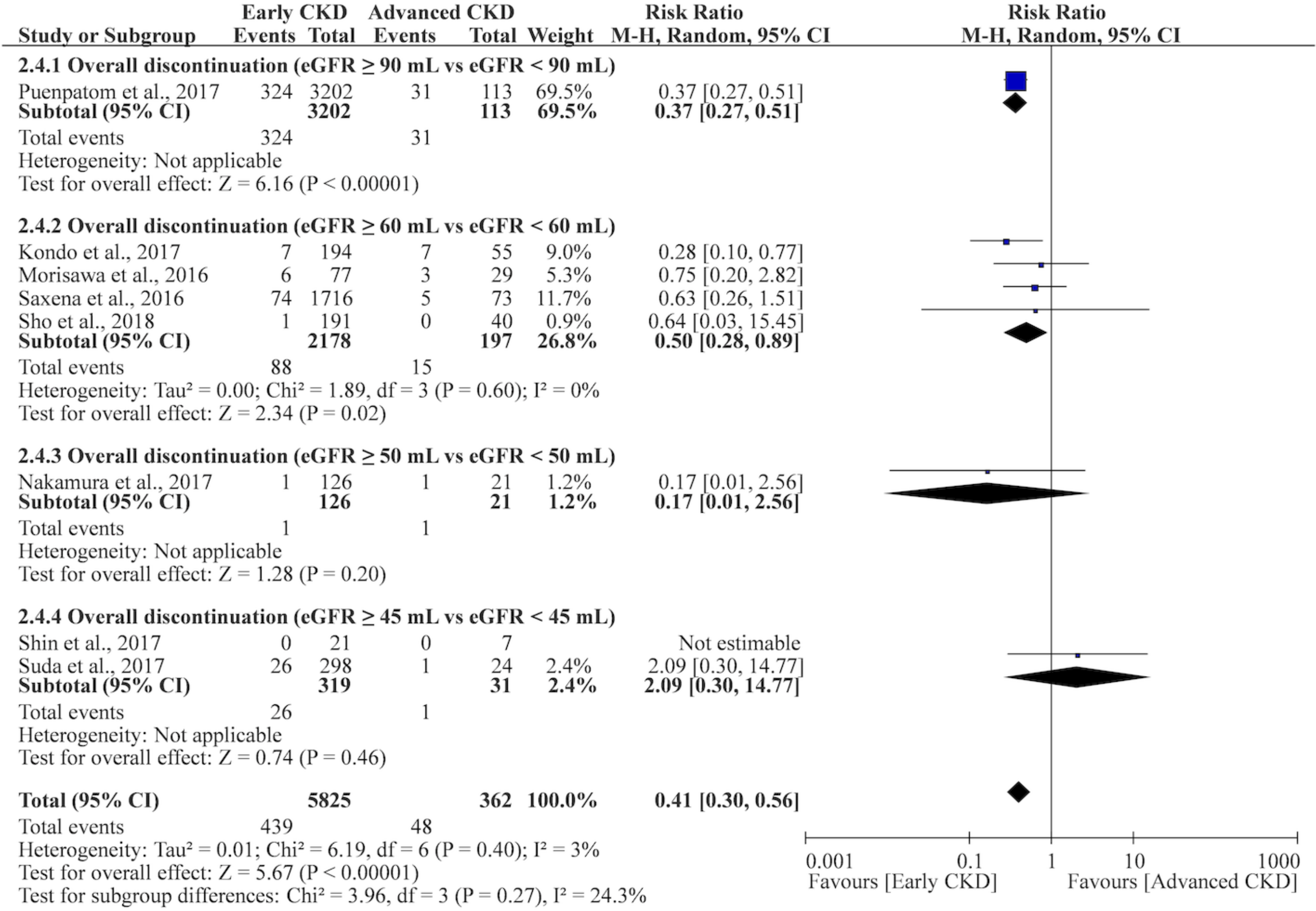

Supplementary Figure 15

Forest plot of discontinuation in fixed-effect model

Study or Subgroup	Early CKD Events	Early CKD Total	Advanced CKD Events	Advanced CKD Total	Weight	Peto Odds Ratio Peto, Fixed, 95% CI
**2.4.1 Overall discontinuation (eGFR ≥ 90 mL vs eGFR < 90 mL)**						
Puenpatom et al., 2017	324	3202	31	113	53.7%	0.16 [0.09, 0.30]
**Subtotal (95% CI)**		**3202**		**113**	**53.7%**	**0.16 [0.09, 0.30]**
Total events	324		31			
Heterogeneity: Not applicable						
Test for overall effect: Z = 5.85 (P < 0.00001)						
**2.4.2 Overall discontinuation (eGFR ≥ 60 mL vs eGFR < 60 mL)**						
Kondo et al., 2017	7	194	7	55	11.7%	0.18 [0.05, 0.66]
Morisawa et al., 2016	6	77	3	29	8.5%	0.72 [0.16, 3.32]
Saxena et al., 2016	74	1716	5	73	15.2%	0.55 [0.18, 1.71]
Sho et al., 2018	1	191	0	40	0.7%	3.35 [0.02, 595.40]
**Subtotal (95% CI)**		**2178**		**197**	**36.2%**	**0.42 [0.20, 0.89]**
Total events	88		15			
Heterogeneity: $Chi^2 = 2.94$, df = 3 (P = 0.40); $I^2 = 0\%$						
Test for overall effect: Z = 2.28 (P = 0.02)						
**2.4.3 Overall discontinuation (eGFR ≥ 50 mL vs eGFR < 50 mL)**						
Nakamura et al., 2017	1	126	1	21	1.3%	0.05 [0.00, 2.82]
**Subtotal (95% CI)**		**126**		**21**	**1.3%**	**0.05 [0.00, 2.82]**
Total events	1		1			
Heterogeneity: Not applicable						
Test for overall effect: Z = 1.45 (P = 0.15)						
**2.4.4 Overall discontinuation (eGFR ≥ 45 mL vs eGFR < 45 mL)**						
Shin et al., 2017	0	21	0	7		Not estimable
Suda et al., 2017	26	298	1	24	8.8%	1.81 [0.40, 8.08]
**Subtotal (95% CI)**		**319**		**31**	**8.8%**	**1.81 [0.40, 8.08]**
Total events	26		1			
Heterogeneity: Not applicable						
Test for overall effect: Z = 0.77 (P = 0.44)						
**Total (95% CI)**		**5825**		**362**	**100.0%**	**0.28 [0.18, 0.44]**
Total events	439		48			
Heterogeneity: $Chi^2 = 13.78$, df = 6 (P = 0.03); $I^2 = 56\%$						
Test for overall effect: Z = 5.59 (P < 0.00001)						
Test for subgroup differences: $Chi^2 = 10.84$, df = 3 (P = 0.01), $I^2 = 72.3\%$						

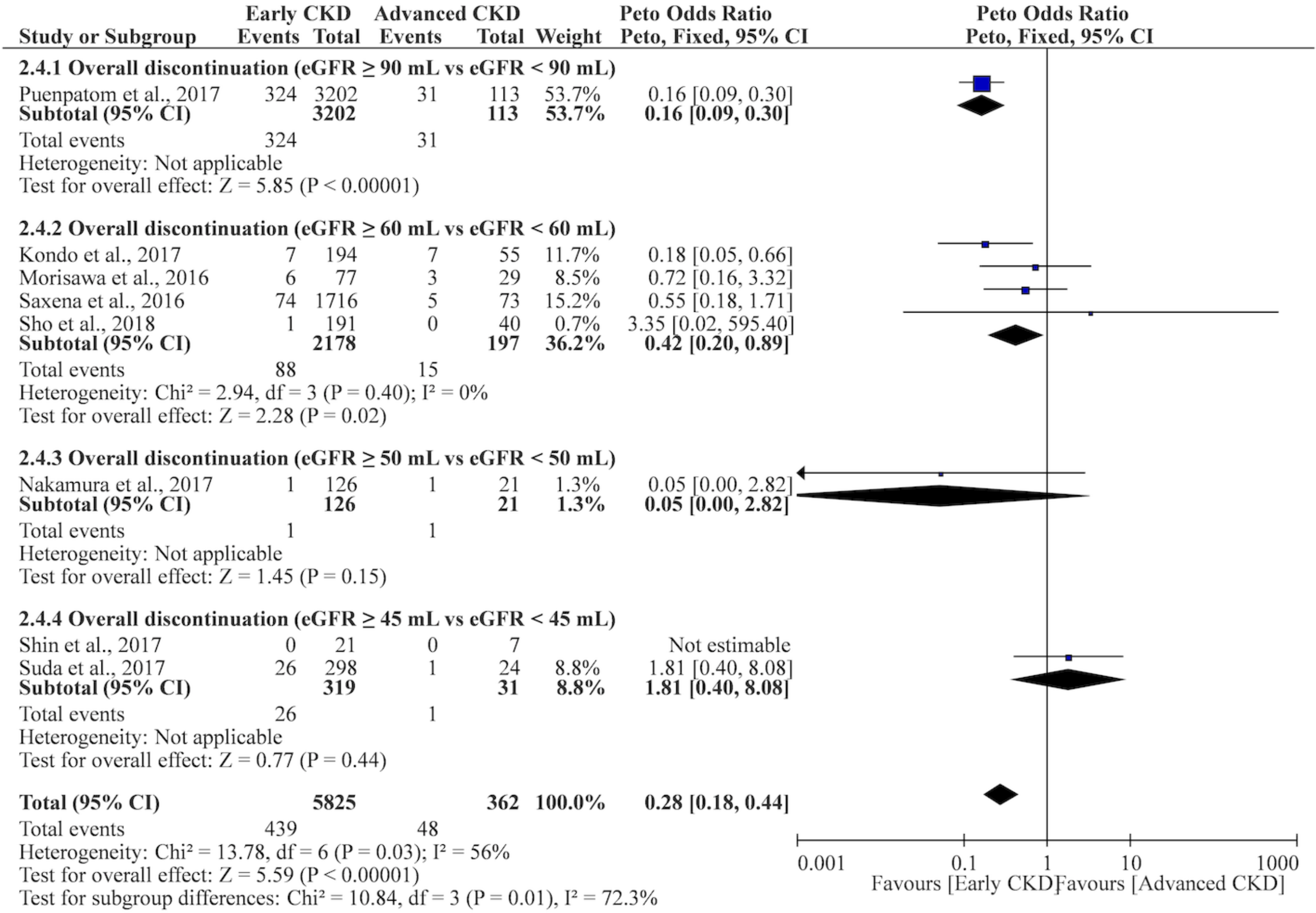

Supplementary Figure 16

Funnel plot of discontinuation

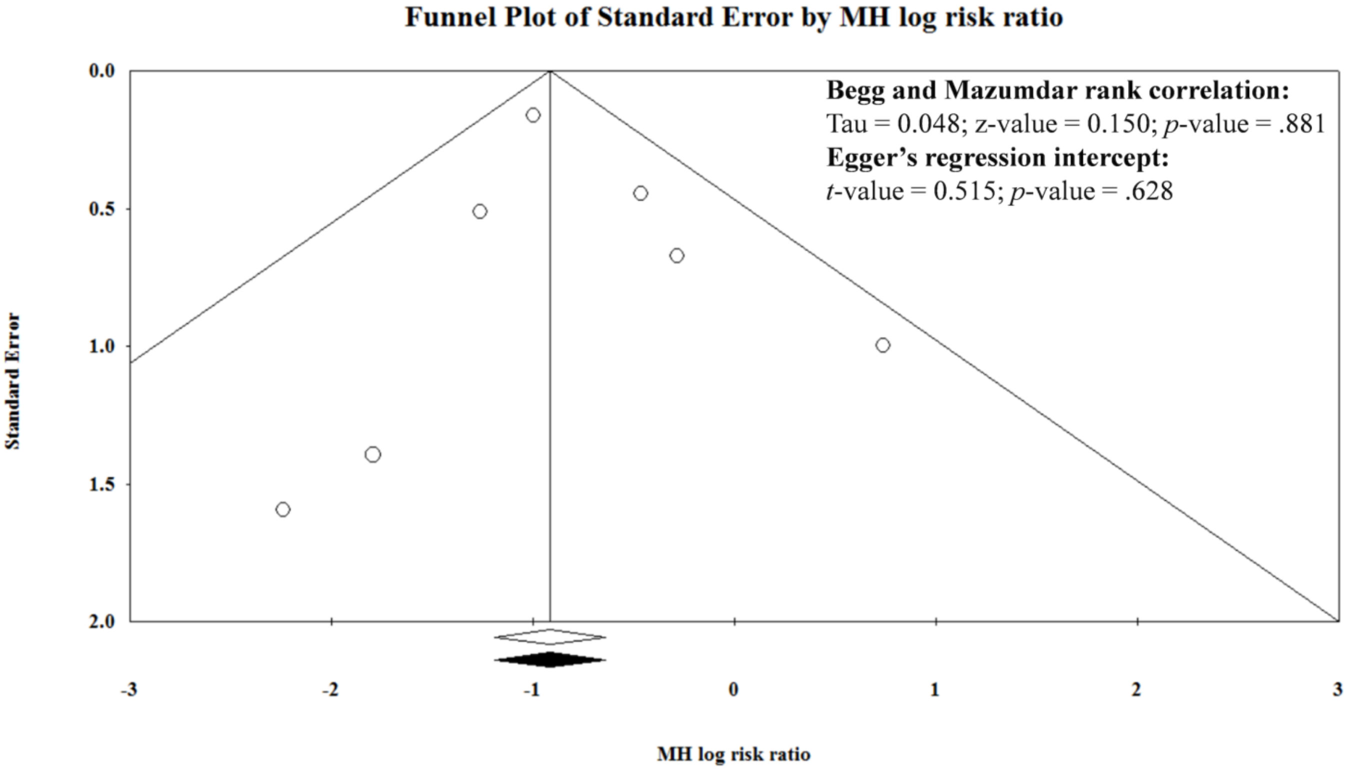

Supplement: Supplementary file 1 [file jcm-07-00314-s001.pdf]
